# Supplementary material for: Colon cancer cell differentiation by sodium butyrate modulates metabolic plasticity of Caco-2 cells via alteration of phosphotransfer network
Source: PLoS One. 2021 Jan 20;16(1):e0245348. doi: 10.1371/journal.pone.0245348 (PMC7817017; doi:10.1371/journal.pone.0245348)
Supplement: S1 Fig — (PPTX) [file pone.0245348.s001.pptx]

## Slide 1
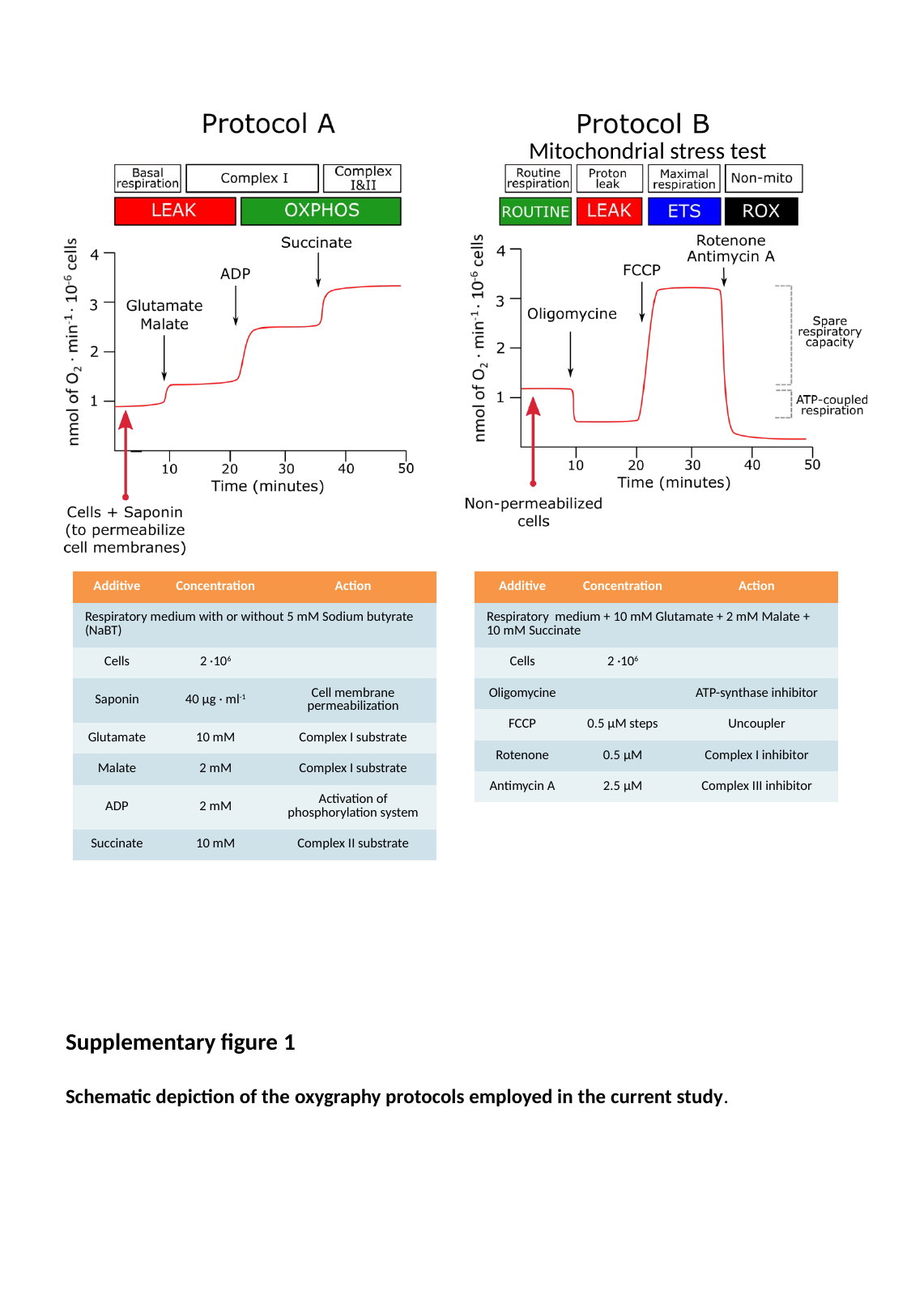

Mitochondrial stress test
| Additive | Concentration | Action |
| --- | --- | --- |
| Respiratory medium with or without 5 mM Sodium butyrate (NaBT) | | |
| Cells | 2 ·106 | |
| Saponin | 40 µg · ml-1 | Cell membrane permeabilization |
| Glutamate | 10 mM | Complex I substrate |
| Malate | 2 mM | Complex I substrate |
| ADP | 2 mM | Activation of phosphorylation system |
| Succinate | 10 mM | Complex II substrate |
| Additive | Concentration | Action |
| --- | --- | --- |
| Respiratory medium + 10 mM Glutamate + 2 mM Malate + 10 mM Succinate | | |
| Cells | 2 ·106 | |
| Oligomycine | | ATP-synthase inhibitor |
| FCCP | 0.5 µM steps | Uncoupler |
| Rotenone | 0.5 µM | Complex I inhibitor |
| Antimycin A | 2.5 µM | Complex III inhibitor |
Supplementary figure 1
Schematic depiction of the oxygraphy protocols employed in the current study.
