# Supplementary material for: Colon cancer cell differentiation by sodium butyrate modulates metabolic plasticity of Caco-2 cells via alteration of phosphotransfer network
Source: PLoS One. 2021 Jan 20;16(1):e0245348. doi: 10.1371/journal.pone.0245348 (PMC7817017; doi:10.1371/journal.pone.0245348)
Supplement: S2 Fig — Bars are SEM (n = 3); ***p<0.001 (Student’s t test). (PPTX) [file pone.0245348.s002.pptx]

## Slide 1
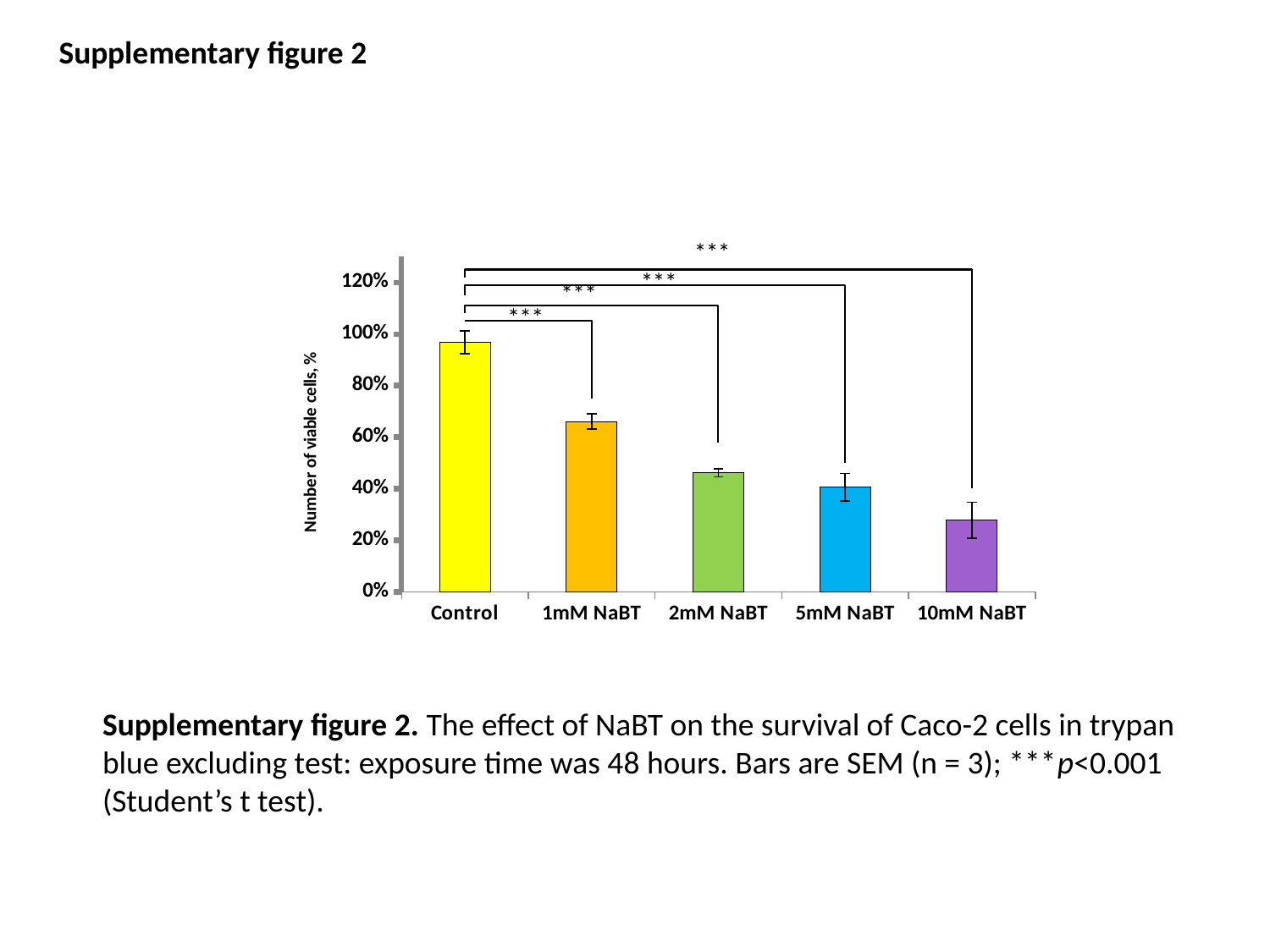

Supplementary figure 2
[unsupported chart]
Supplementary figure 2. The effect of NaBT on the survival of Caco-2 cells in trypan blue excluding test: exposure time was 48 hours. Bars are SEM (n = 3); ***p<0.001 (Student’s t test).
