# Supplementary figures and images for: Colon cancer cell differentiation by sodium butyrate modulates metabolic plasticity of Caco-2 cells via alteration of phosphotransfer network
Source: PLoS One. 2021 Jan 20;16(1):e0245348. doi: 10.1371/journal.pone.0245348 (PMC7817017; doi:10.1371/journal.pone.0245348)

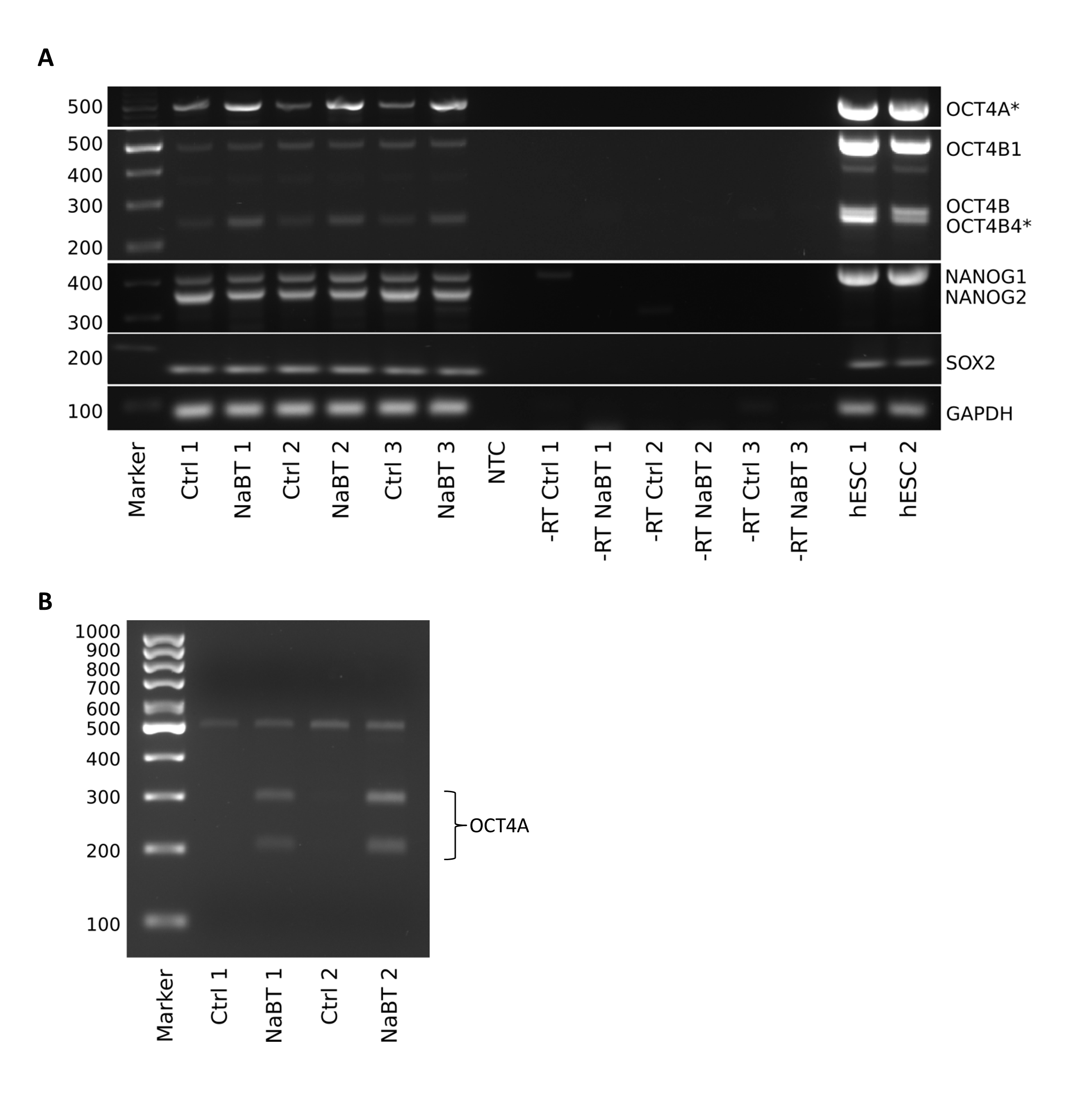

Supplement: S3 Fig — (A): Detection of main OCT4 spliced variants, transcripts from NANOG1, NANOG2 and SOX2. GAPDH was used for loading control. OCT4A* primers can also amplify transcripts from OCT4 pseudogenes. OCT4B/B1 primers allow detection of OCT4 variant OCT4B4* (B): Restriction analysis of OCT4A* PCR product with ApaI showing 204 bp and 291bp fragments in the presence of OCT4A and 496bp product representative of OCT4 pseudogenes. (TIF) [file pone.0245348.s003.tif]

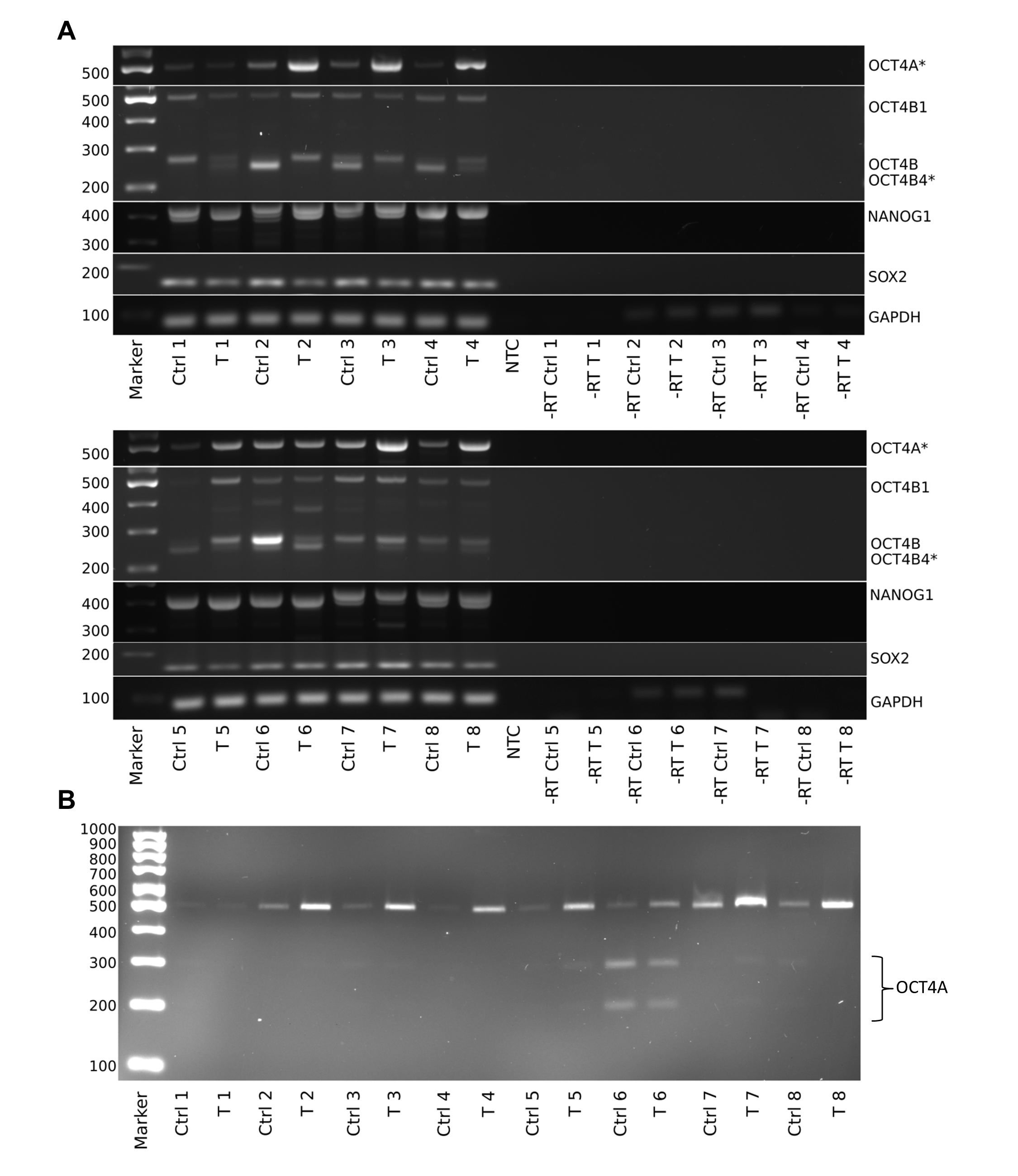

Supplement: S4 Fig — (A): Detection of main OCT4 spliced variants, transcripts from NANOG1, NANOG2 and SOX2. GAPDH was used for loading control. OCT4A* primers can also amplify transcripts from OCT4 pseudogenes. OCT4B/B1 primers allow detection of OCT4 variant OCT4B4* (B): Restriction analysis of OCT4A* PCR product with ApaI showing 204 bp and 291bp fragments in the presence of OCT4A and 496bp product representative of OCT4 pseudogenes. (TIF) [file pone.0245348.s004.tif]
