# Supplementary material for: Colon cancer cell differentiation by sodium butyrate modulates metabolic plasticity of Caco-2 cells via alteration of phosphotransfer network
Source: PLoS One. 2021 Jan 20;16(1):e0245348. doi: 10.1371/journal.pone.0245348 (PMC7817017; doi:10.1371/journal.pone.0245348)
Supplement: S5 Fig — OCT4B1 is highly expressed in human embryonic stem cells (hESC) together with both OCT4B and OCT4B4 transcripts. Caco-2 cells express OCT4B1 and OCT4B4, but not the OCT4B variant. In colorectal tumor and respective control samples the expression of OCT4B4 and OCT4B varies while OCT4B1 is expressed on low level. (PPTX) [file pone.0245348.s005.pptx]

## Slide 1
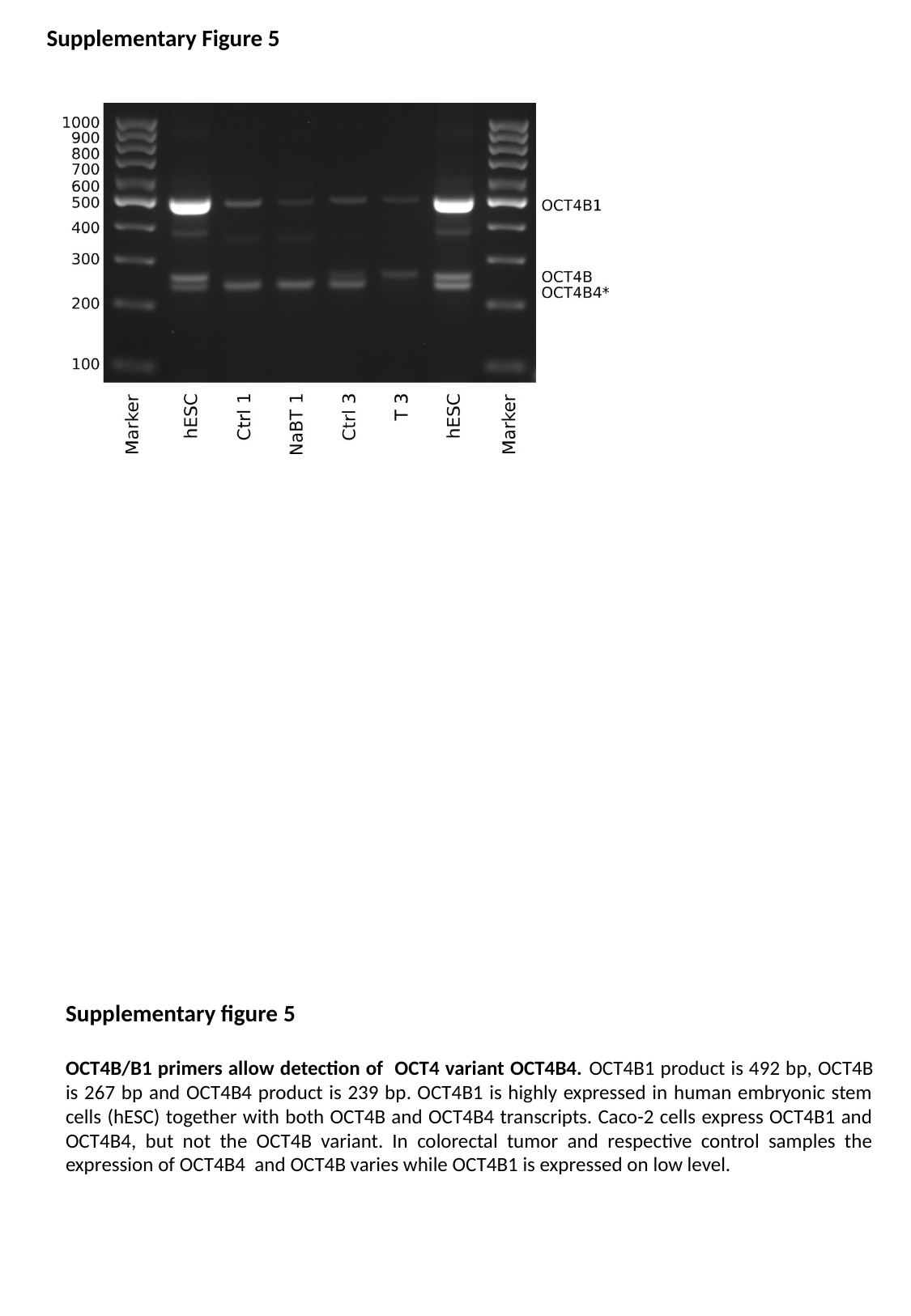

Supplementary Figure 5
### Chart
| Category |
|---|Supplementary figure 5
OCT4B/B1 primers allow detection of OCT4 variant OCT4B4. OCT4B1 product is 492 bp, OCT4B is 267 bp and OCT4B4 product is 239 bp. OCT4B1 is highly expressed in human embryonic stem cells (hESC) together with both OCT4B and OCT4B4 transcripts. Caco-2 cells express OCT4B1 and OCT4B4, but not the OCT4B variant. In colorectal tumor and respective control samples the expression of OCT4B4 and OCT4B varies while OCT4B1 is expressed on low level.
