# Supplementary material for: Colon cancer cell differentiation by sodium butyrate modulates metabolic plasticity of Caco-2 cells via alteration of phosphotransfer network
Source: PLoS One. 2021 Jan 20;16(1):e0245348. doi: 10.1371/journal.pone.0245348 (PMC7817017; doi:10.1371/journal.pone.0245348)
Supplement: S6 Fig — (A): Specific citrate synthase activity. (B,C): Mitochondrial membrane potential was assayed by TMRE and MitoTracker Red staining using fluorescence microplate reader. Data presented as mean ± SEM (n = 5). NaBT–sodium butyrate. (PPTX) [file pone.0245348.s006.pptx]

## Slide 1
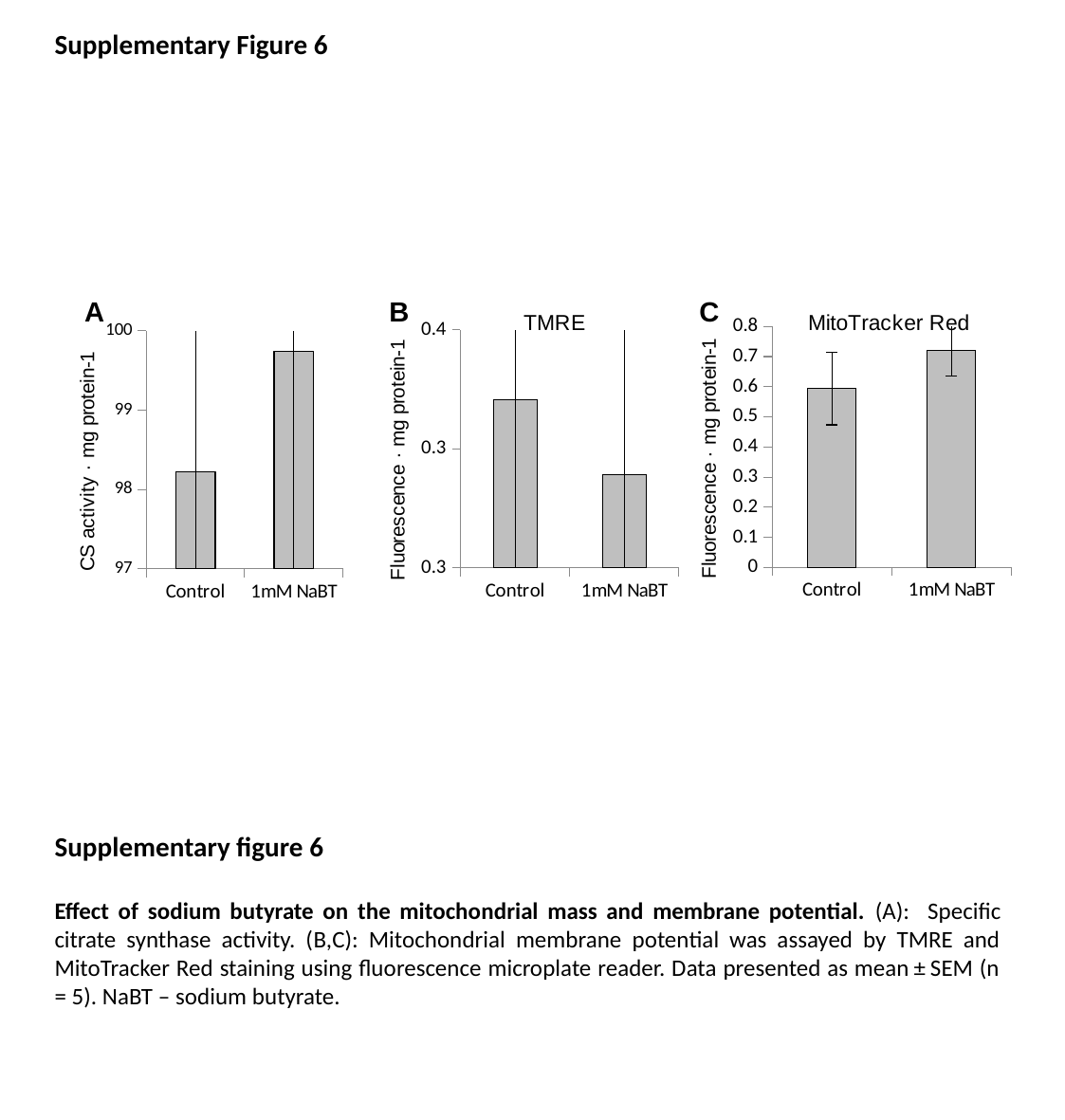

Supplementary Figure 6
### Chart
| Category | |
|---|---|
| Control | 98.22453703703701 |
| 1mM NaBT | 99.74162161435333 |
### Chart
| Category | |
|---|---|
| Control | 0.3481583333333334 |
| 1mM NaBT | 0.33569166666666855 |
### Chart
| Category | |
|---|---|
| Control | 0.593359884313069 |
| 1mM NaBT | 0.7195882895909568 |Supplementary figure 6
Effect of sodium butyrate on the mitochondrial mass and membrane potential. (A): Specific citrate synthase activity. (B,C): Mitochondrial membrane potential was assayed by TMRE and MitoTracker Red staining using fluorescence microplate reader. Data presented as mean ± SEM (n = 5). NaBT – sodium butyrate.
