# Supplementary material for: Colon cancer cell differentiation by sodium butyrate modulates metabolic plasticity of Caco-2 cells via alteration of phosphotransfer network
Source: PLoS One. 2021 Jan 20;16(1):e0245348. doi: 10.1371/journal.pone.0245348 (PMC7817017; doi:10.1371/journal.pone.0245348)
Supplement: S1 Table — (DOCX) [file pone.0245348.s007.docx]

**Supplementary Table 1.** Changes in the AK isoforms in cancer cells and tissues described in the literature

| **Enzyme isoform** | **Type of cancer** | **Status in tumor** | **Experimental model** | **Ref.** |
| --- | --- | --- | --- | --- |
| **AK1** | Transformed embryonic fibroblasts | ↓ | ras^V12^/E1A-transformed primary mouse embryonic fibroblasts | (Vasseur, Malicet et al. 2005) |
| **AK1** | Breast cancer | ↑ | Tissue samples | (Klepinin, Ounpuu et al. 2016) |
| **AK2** | Breast cancer | ↑ | Estrogen receptor negative breast cancer | (Speers, Tsimelzon et al. 2009) |
| **AK2** | Breast cancer | ↑ | Tissue samples | (Klepinin, Ounpuu et al. 2016) |
| **AK2** | Breast cancer | ↓ | Breast cancer cell lines | (Kim, Lee et al. 2014) |
| **AK4** | Lung cancer | ↑ | Tissue samples  Various cell lines | (Jan, Tsai et al. 2012) |
| **AK6** | Breast cancer  Colon cancer | ↑ | Colon adenocarcinoma and breast cancer tissues | (Bai, Zhang et al. 2016) |
| **AK6** | Colon cancer | ↑ | CSCs from CRC tissues | (Ji, Yang et al. 2017) |
| **AK** | Colon cancer | ↑ | Tissue samples | (Chekulayev, Mado et al. 2015) |
| **AK** | Colon cancer | ↑ | Tissue samples | (Kaldma, Klepinin et al. 2014) |
| **AK** | Lung cancer | ↓ | Tissue samples | (Balinsky, Greengard et al. 1984) |

**References**

Bai, D., J. Zhang, T. Li, R. Hang, Y. Liu, Y. Tian, D. Huang, L. Qu, X. Cao, J. Ji and X. Zheng (2016). "The ATPase hCINAP regulates 18S rRNA processing and is essential for embryogenesis and tumour growth." Nature communications **7**: 12310.

Balinsky, D., O. Greengard, E. Cayanis and J. F. Head (1984). "Enzyme activities and isozyme patterns in human lung tumors." Cancer Res **44**(3): 1058-1062.

Chekulayev, V., K. Mado, I. Shevchuk, A. Koit, A. Kaldma, A. Klepinin, N. Timohhina, K. Tepp, M. Kandashvili, L. Ounpuu, K. Heck, L. Truu, A. Planken, V. Valvere and T. Kaambre (2015). "Metabolic remodeling in human colorectal cancer and surrounding tissues: alterations in regulation of mitochondrial respiration and metabolic fluxes." Biochemistry and Biophysics Reports **4**: 111-125.

Jan, Y. H., H. Y. Tsai, C. J. Yang, M. S. Huang, Y. F. Yang, T. C. Lai, C. H. Lee, Y. M. Jeng, C. Y. Huang, J. L. Su, Y. J. Chuang and M. Hsiao (2012). "Adenylate kinase-4 is a marker of poor clinical outcomes that promotes metastasis of lung cancer by downregulating the transcription factor ATF3." Cancer Res **72**(19): 5119-5129.

Ji, Y., C. Yang, Z. Tang, Y. Yang, Y. Tian, H. Yao, X. Zhu, Z. Zhang, J. Ji and X. Zheng (2017). "Adenylate kinase hCINAP determines self-renewal of colorectal cancer stem cells by facilitating LDHA phosphorylation." Nature Communications **8**: 15308.

Kaldma, A., A. Klepinin, V. Chekulayev, K. Mado, I. Shevchuk, N. Timohhina, K. Tepp, M. Kandashvili, M. Varikmaa, A. Koit, M. Planken, K. Heck, L. Truu, A. Planken, V. Valvere, E. Rebane and T. Kaambre (2014). "An in situ study of bioenergetic properties of human colorectal cancer: the regulation of mitochondrial respiration and distribution of flux control among the components of ATP synthasome." Int J Biochem Cell Biol **55**: 171-186.

Kim, H., H. J. Lee, Y. Oh, S. G. Choi, S. H. Hong, H. J. Kim, S. Y. Lee, J. W. Choi, D. Su Hwang, K. S. Kim, H. J. Kim, J. Zhang, H. J. Youn, D. Y. Noh and Y. K. Jung (2014). "The DUSP26 phosphatase activator adenylate kinase 2 regulates FADD phosphorylation and cell growth." Nat Commun **5**: 3351.

Klepinin, A., L. Ounpuu, R. Guzun, V. Chekulayev, N. Timohhina, K. Tepp, I. Shevchuk, U. Schlattner and T. Kaambre (2016). "Simple oxygraphic analysis for the presence of adenylate kinase 1 and 2 in normal and tumor cells." J Bioenerg Biomembr **48**(5): 531-548.

Speers, C., A. Tsimelzon, K. Sexton, A. M. Herrick, C. Gutierrez, A. Culhane, J. Quackenbush, S. Hilsenbeck, J. Chang and P. Brown (2009). "Identification of Novel Kinase Targets for the Treatment of Estrogen Receptor-Negative Breast Cancer." Clinical Cancer Research **15**: 6327-6340.

Vasseur, S., C. Malicet, E. L. Calvo, J. Dagorn and J. L. Iovanna (2005). "Gene expression profiling of tumours derived from rasV12/E1A-transformed mouse embryonic fibroblasts to identify genes required for tumour development." Molecular Cancer **4**: 4.
