# Supplementary material for: Colon cancer cell differentiation by sodium butyrate modulates metabolic plasticity of Caco-2 cells via alteration of phosphotransfer network
Source: PLoS One. 2021 Jan 20;16(1):e0245348. doi: 10.1371/journal.pone.0245348 (PMC7817017; doi:10.1371/journal.pone.0245348)
Supplement: S2 Table — (DOCX) [file pone.0245348.s008.docx]

**Supplementary Table 2.** Primers and product sizes in quantitative RT-PCR:

| **SOX2** | F: CATGCACCGCTACGACG  R: CGGACTTGACCACCGAAC | 152 bp | Park et al., 2012, Cell Death & Differentiation |
| --- | --- | --- | --- |
| **OCT4A** | F: CTGGAGCAAAACCCGGAGG  R: CCTCAAAGCGGCAGATGGTC | 181 bp | Designed by us |
| **GAPDH** | F: TGCACCACCAACTGCTTAGC  R: GGCATGGACTGTGGTCATGAG | 87 bp | Hruz et al., 2011, BMC Genomics |
