# Supplementary material for: Colon cancer cell differentiation by sodium butyrate modulates metabolic plasticity of Caco-2 cells via alteration of phosphotransfer network
Source: PLoS One. 2021 Jan 20;16(1):e0245348. doi: 10.1371/journal.pone.0245348 (PMC7817017; doi:10.1371/journal.pone.0245348)
Supplement: S3 Table — (DOCX) [file pone.0245348.s009.docx]

**Supplementary Table 3.** Quantitative RT-PCR amplification program:

| 95 °C – 600 s | |
| --- | --- |
| 95 °C – 15 s |  |
|  | 40x |
| 61 °C – 60 s |  |
