# Supplementary material for: Colon cancer cell differentiation by sodium butyrate modulates metabolic plasticity of Caco-2 cells via alteration of phosphotransfer network
Source: PLoS One. 2021 Jan 20;16(1):e0245348. doi: 10.1371/journal.pone.0245348 (PMC7817017; doi:10.1371/journal.pone.0245348)
Supplement: S4 Table — (DOCX) [file pone.0245348.s010.docx]

**Supplementary Table 4.** Primers and product sizes in semi-quantitative RT-PCR:

| **SOX2** | F: CATGCACCGCTACGACG  R: CGGACTTGACCACCGAAC | 152 bp | Park et al., 2012, Cell Death & Differentiation |
| --- | --- | --- | --- |
| **NANOG1/2** | F: GCCTCCAGCAGATGCAAGAAC  R: GCAGGAGAATTTGGCTGGAAC | 418 bp / 370 bp | Eberle et al., 2010, Nucleic Acids Res |
| **OCT4A** | F: CTTCTCGCCCCCTCCAGGT  R: AAATAGAACCCCCAGGGTGAGC | 496 bp | Atlasi et al., 2008, Stem Cells |
| **OCT4B/B1** | F: AGACTATTCCTTGGGGCCACAC  R: CTCAAAGCGGCAGATGGTCG | 267 bp / 492 bp | Atlasi et al., 2008, Stem Cells |
| **GAPDH** | F: TGCACCACCAACTGCTTAGC  R: GGCATGGACTGTGGTCATGAG | 87 bp | Hruz et al., 2011, BMC Genomics |
