# Supplementary material for: Colon cancer cell differentiation by sodium butyrate modulates metabolic plasticity of Caco-2 cells via alteration of phosphotransfer network
Source: PLoS One. 2021 Jan 20;16(1):e0245348. doi: 10.1371/journal.pone.0245348 (PMC7817017; doi:10.1371/journal.pone.0245348)
Supplement: S5 Table — (DOCX) [file pone.0245348.s011.docx]

**Supplementary Table 5.** Semi-quantitative RT-PCR amplification programs:

| **SOX2** |  | | **NANOG1/2** | | **OCT4A** | | **OCT4B/B1** | | **GAPDH** |  | |
| --- | --- | --- | --- | --- | --- | --- | --- | --- | --- | --- | --- |
| 94 °C – 120 s | | | 94 °C – 120 s | | 94 °C – 120 s | | 94 °C – 120 s | | 94 °C – 120 s | | |
| 94 °C – 30 s | |  | 94 °C – 30 s |  | 94 °C – 30 s |  | 94 °C – 30 s |  | 94 °C – 30 s | |  |
| 59 °C – 30 s | | 40x | 58 °C – 30 s | 40x | 64 °C – 30 s | 35x | 64 °C – 30 s | 32x | 63 °C – 30 s | | 35x |
| 72 °C – 30 s | |  | 72 °C – 90 s |  | 72 °C – 35 s |  | 72 °C – 35 s |  | 72 °C – 30 s | |  |
| 72 °C – 5 min | | | 72 °C – 5 min | | 72 °C – 10 min | | 72 °C – 10 min | | 72 °C – 5 min | | |
