# Supplementary material for: Colon cancer cell differentiation by sodium butyrate modulates metabolic plasticity of Caco-2 cells via alteration of phosphotransfer network
Source: PLoS One. 2021 Jan 20;16(1):e0245348. doi: 10.1371/journal.pone.0245348 (PMC7817017; doi:10.1371/journal.pone.0245348)
Supplement: S1 File — (PDF) [file pone.0245348.s012.pdf]

Microsynth

Technical Datasheet

Order Id 639599 *Order Date* 28.06.2019

Mycoplasma_F *Oligo Id* **3508939**

Type	DNA	*Synthesis scale*	Genomics	*Molecular weight*	7724.0 g/mol	*Amount*	12.55 OD
5' Modification	NONE	*Purification*	Desalted	*Ext. coefficient*	263.0 1/(mM·cm)		47.7 nmol
3' Modification	NONE	*Length*	25 nt	*Tm (50 mM NaCl)*	65.8 °C		368.5 µg
No internal modifications				*Tm (NN-Method)*	56.5 °C	*Volume for 100 µM*	477.1 µl

5'-GGG AGC AAA CAG GAT TAG ATA CCC T-3'

Mycoplasma_R *Oligo Id* **3508940**

Type	DNA	*Synthesis scale*	Genomics	*Molecular weight*	8121.3 g/mol	*Amount*	13.43 OD
5' Modification	NONE	*Purification*	Desalted	*Ext. coefficient*	237.9 1/(mM·cm)		56.5 nmol
3' Modification	NONE	*Length*	27 nt	*Tm (50 mM NaCl)*	68.2 °C		458.7 µg
No internal modifications				*Tm (NN-Method)*	59.2 °C	*Volume for 100 µM*	564.8 µl

5'-TGC ACC ATC TGT CAC TCT GTT AAC CTC-3'
