# Supplementary material for: Colon cancer cell differentiation by sodium butyrate modulates metabolic plasticity of Caco-2 cells via alteration of phosphotransfer network
Source: PLoS One. 2021 Jan 20;16(1):e0245348. doi: 10.1371/journal.pone.0245348 (PMC7817017; doi:10.1371/journal.pone.0245348)

S3A Fig :

OCT4A*

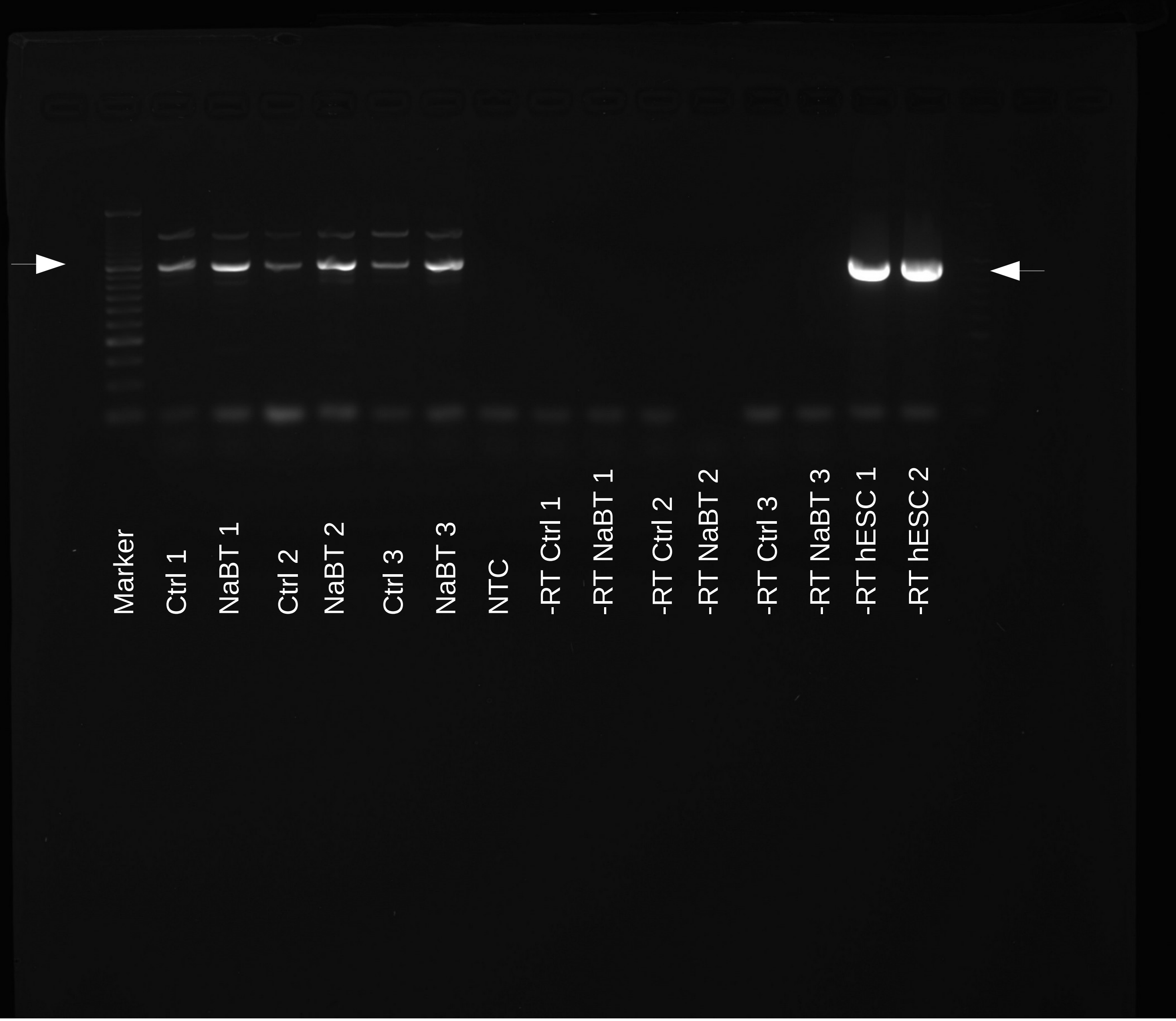

S3A Fig :

OCT4B1
OCT4B
OCT4B4

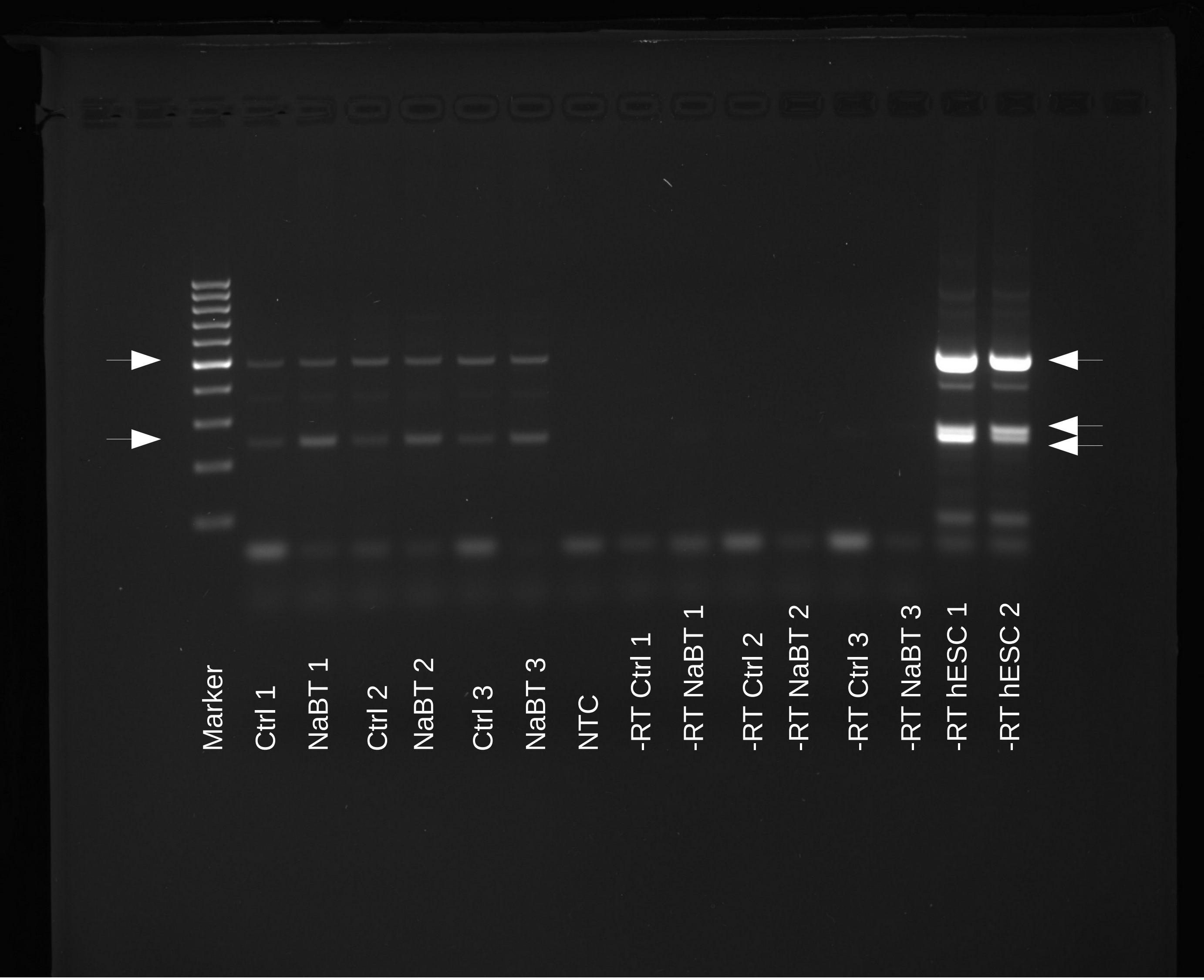

S3A Fig :

NANOG1
NANOG2

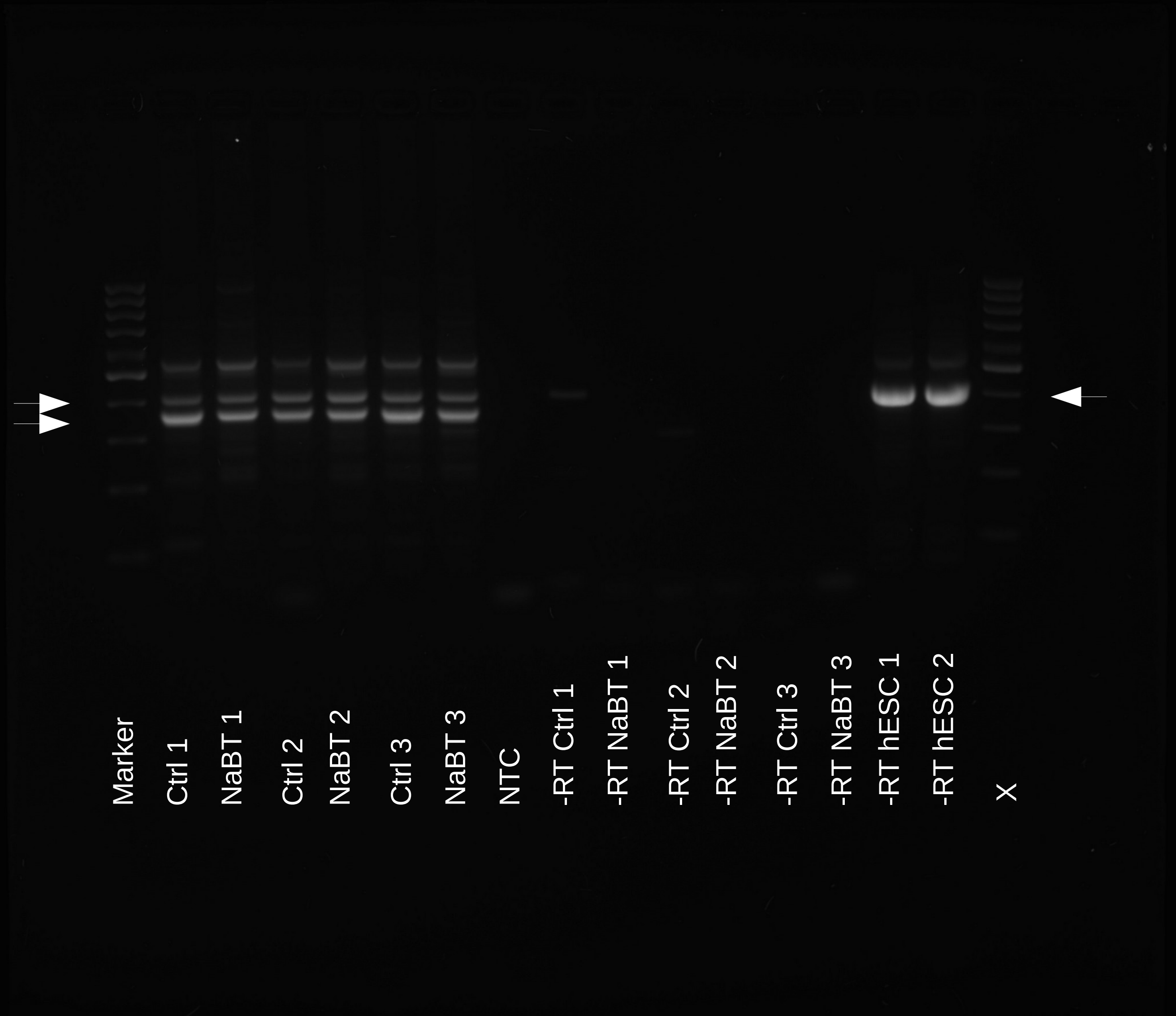

S3A Fig :

SOX2

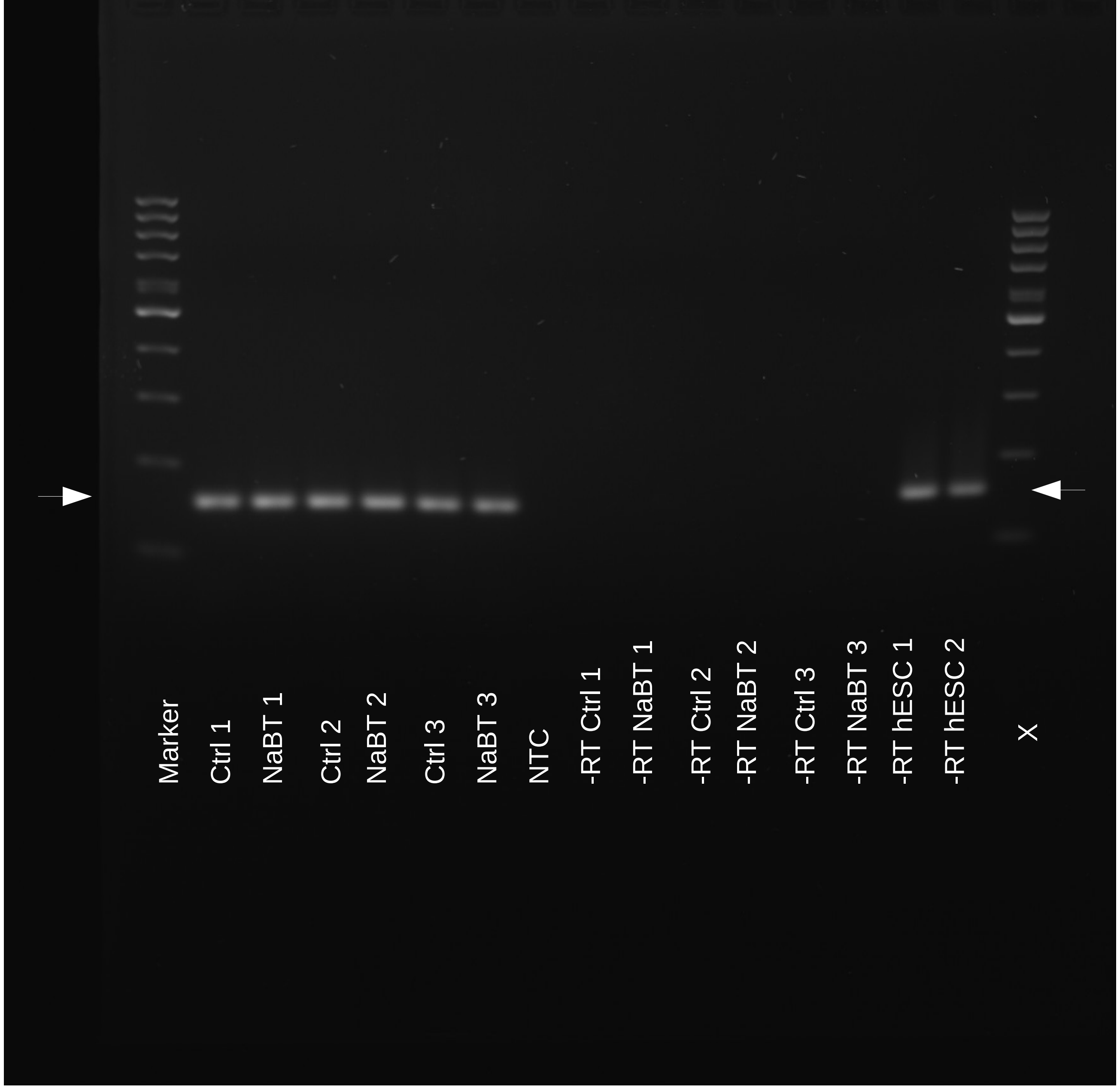

Marker
Ctrl 1
NaBT 1
Ctrl 2
NaBT 2
Ctrl 3
NaBT 3
NTC
-RT Ctrl 1
-RT NaBT 1
-RT Ctrl 2
-RT NaBT 2
-RT Ctrl 3
-RT NaBT 3
-RT hESC 1
-RT hESC 2
X

S3A Fig :

GAPDH

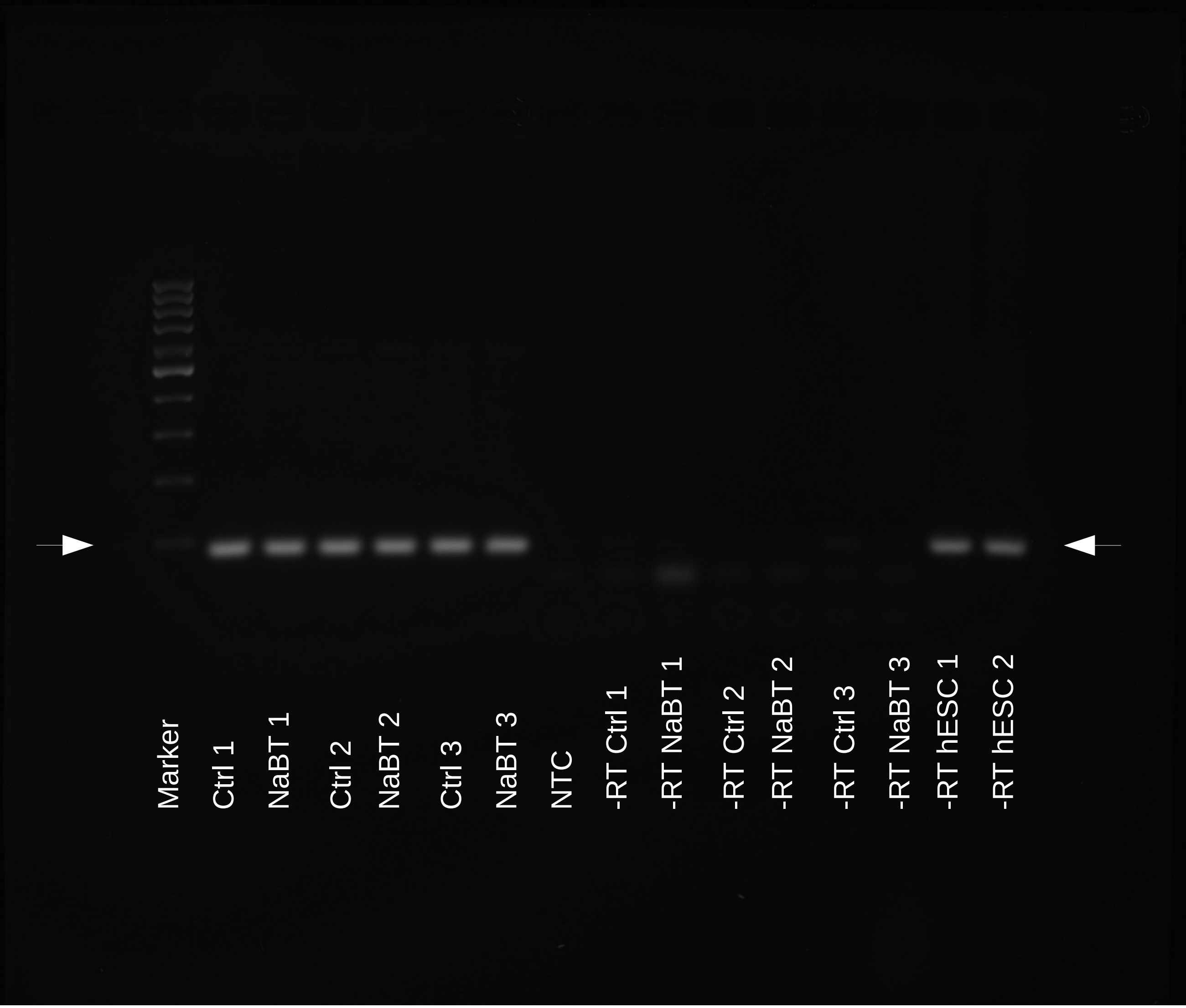

S3B Fig :

OCT4 pseudogene,
OCT4A

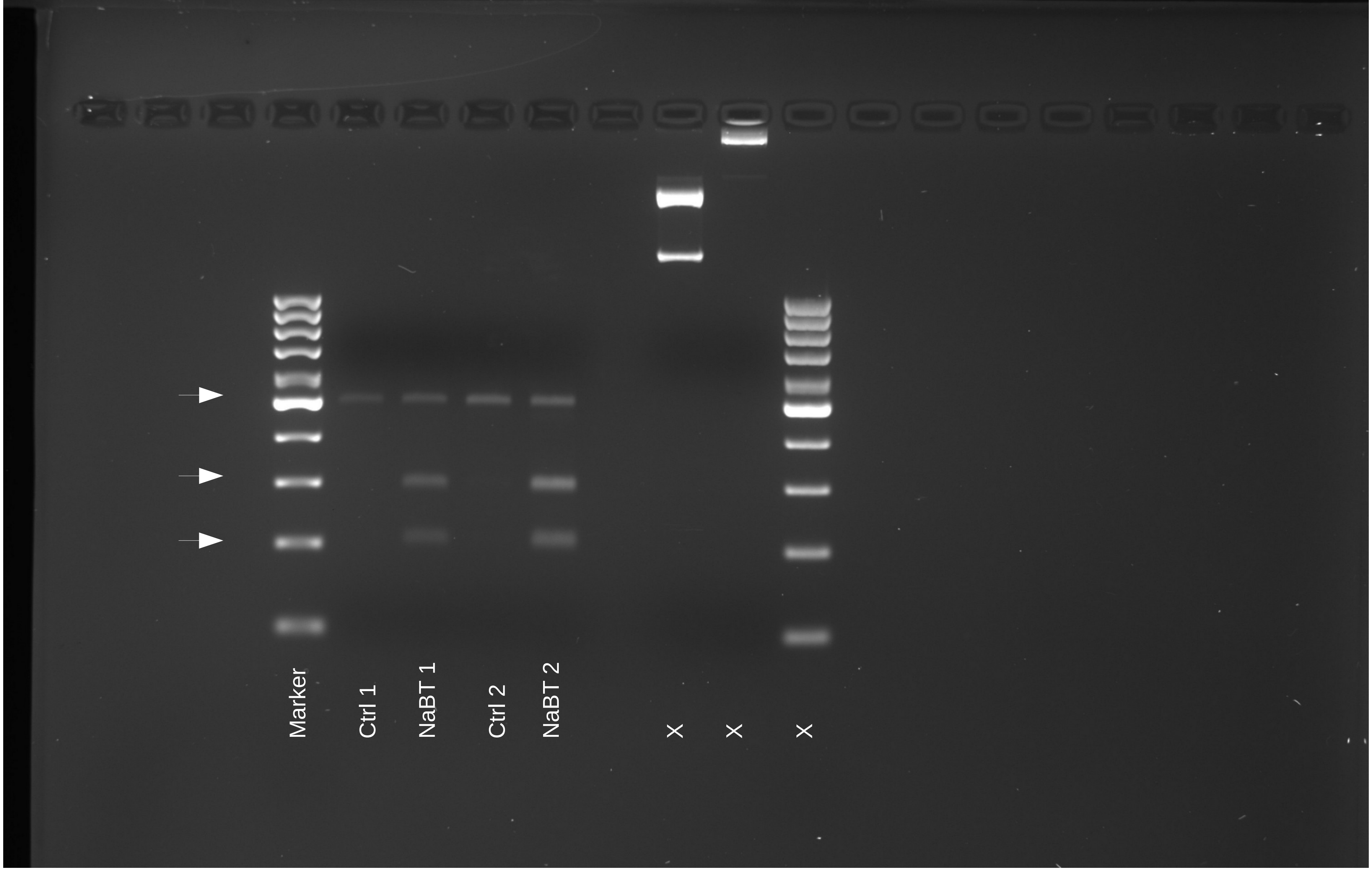

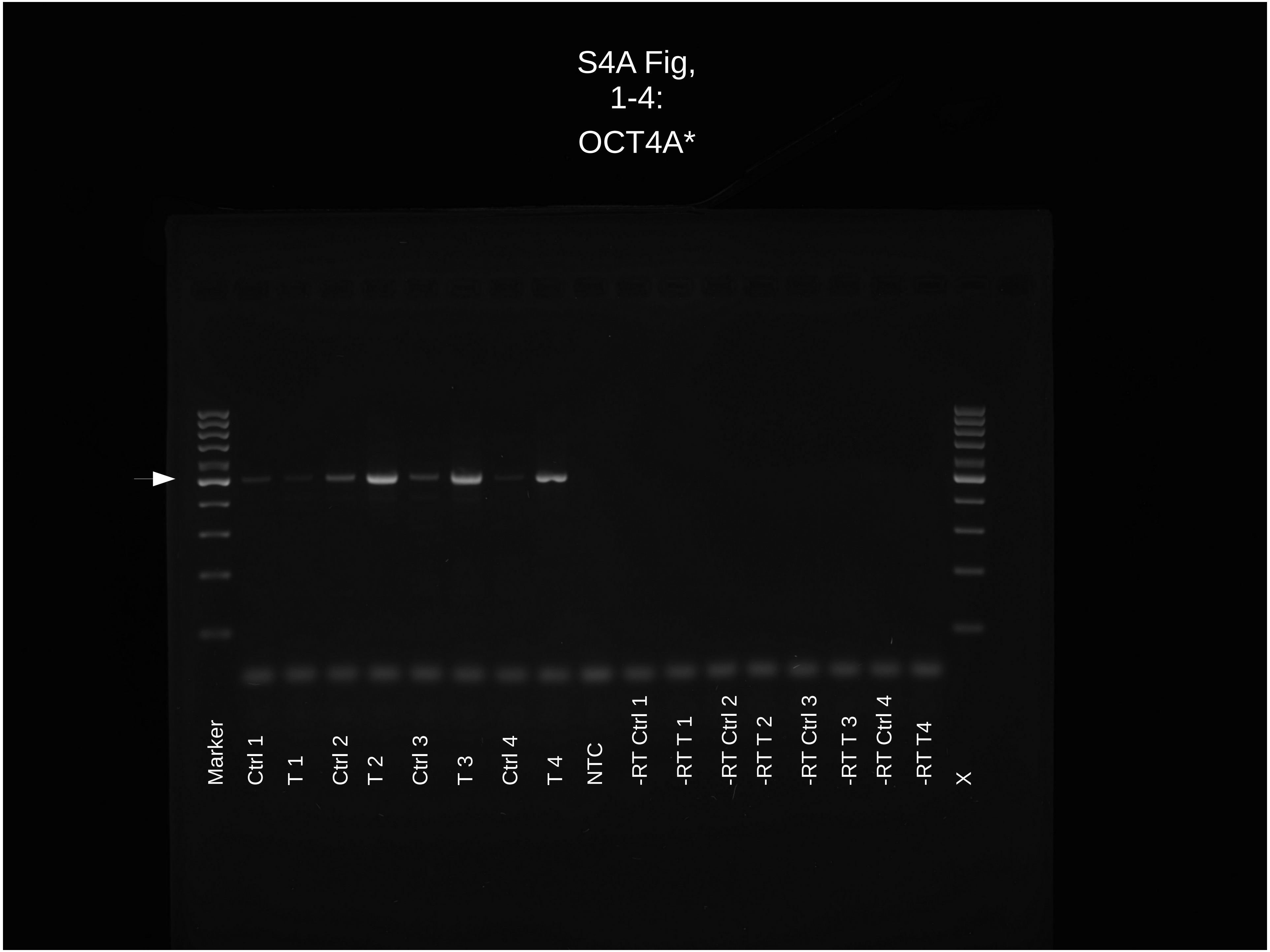
S4A Fig,
1-4:
OCT4A*
Marker
Ctrl 1
T 1
Ctrl 2
T 2
Ctrl 3
T 3
Ctrl 4
T 4
NTC
-RT Ctrl 1
-RT T 1
-RT Ctrl 2
-RT T 2
-RT Ctrl 3
-RT T 3
-RT Ctrl 4
-RT T4
X

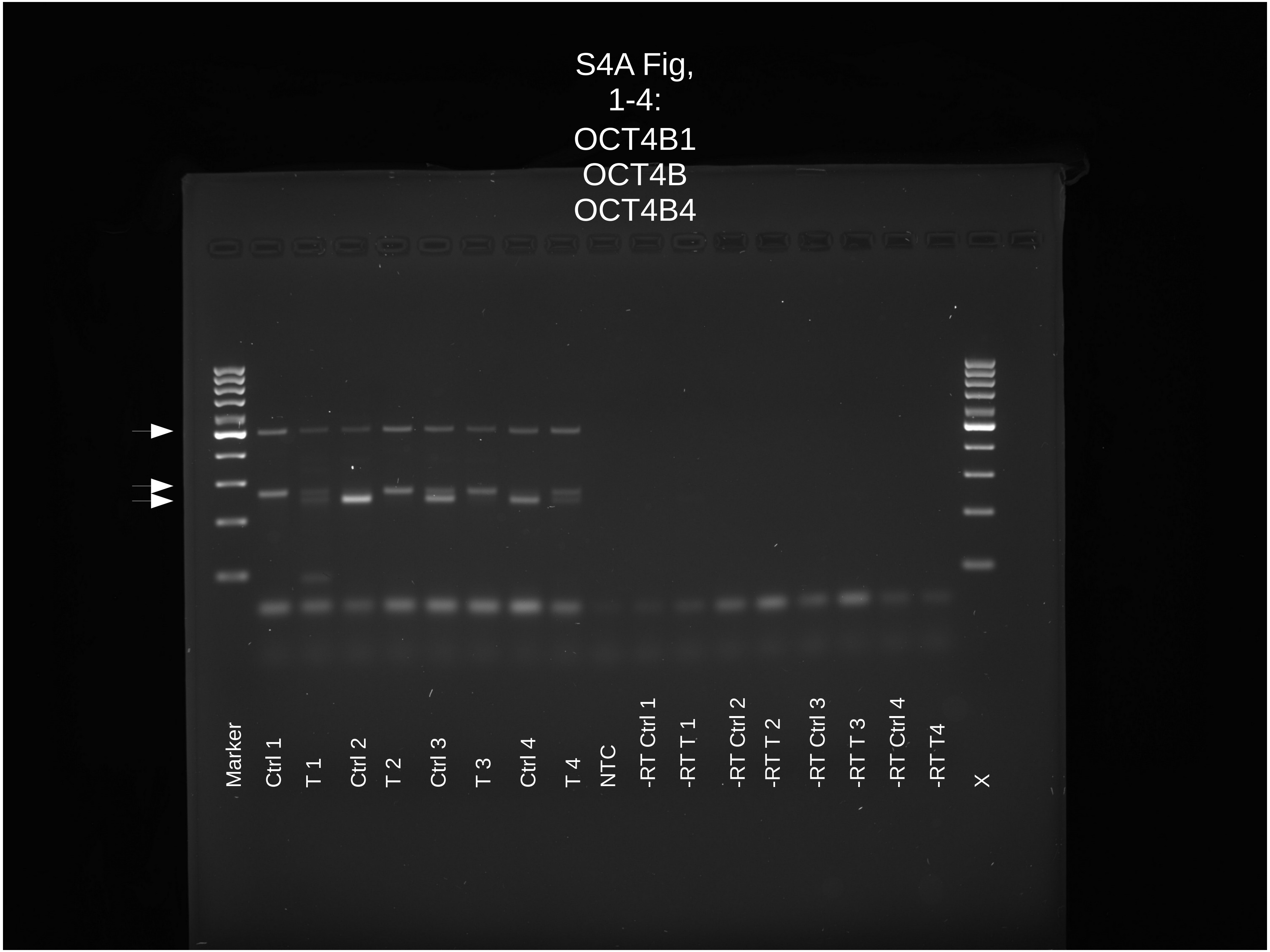
S4A Fig,
1-4:
OCT4B1
OCT4B
OCT4B4
Marker
Ctrl 1
T 1
Ctrl 2
T 2
Ctrl 3
T 3
Ctrl 4
T 4
NTC
-RT Ctrl 1
-RT T 1
-RT Ctrl 2
-RT T 2
-RT Ctrl 3
-RT T 3
-RT Ctrl 4
-RT T4
X

S4A Fig,
1-4:
NANOG1

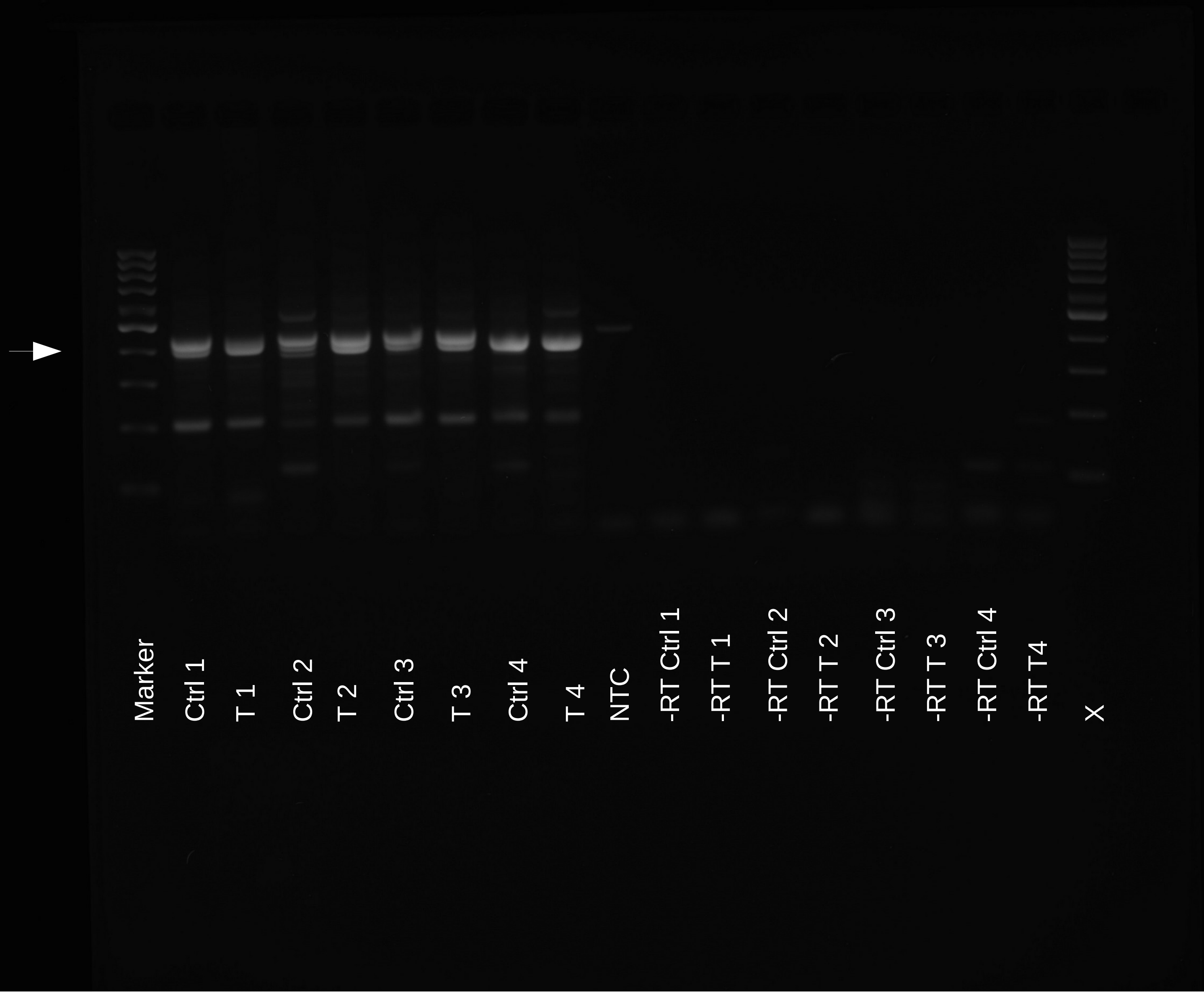

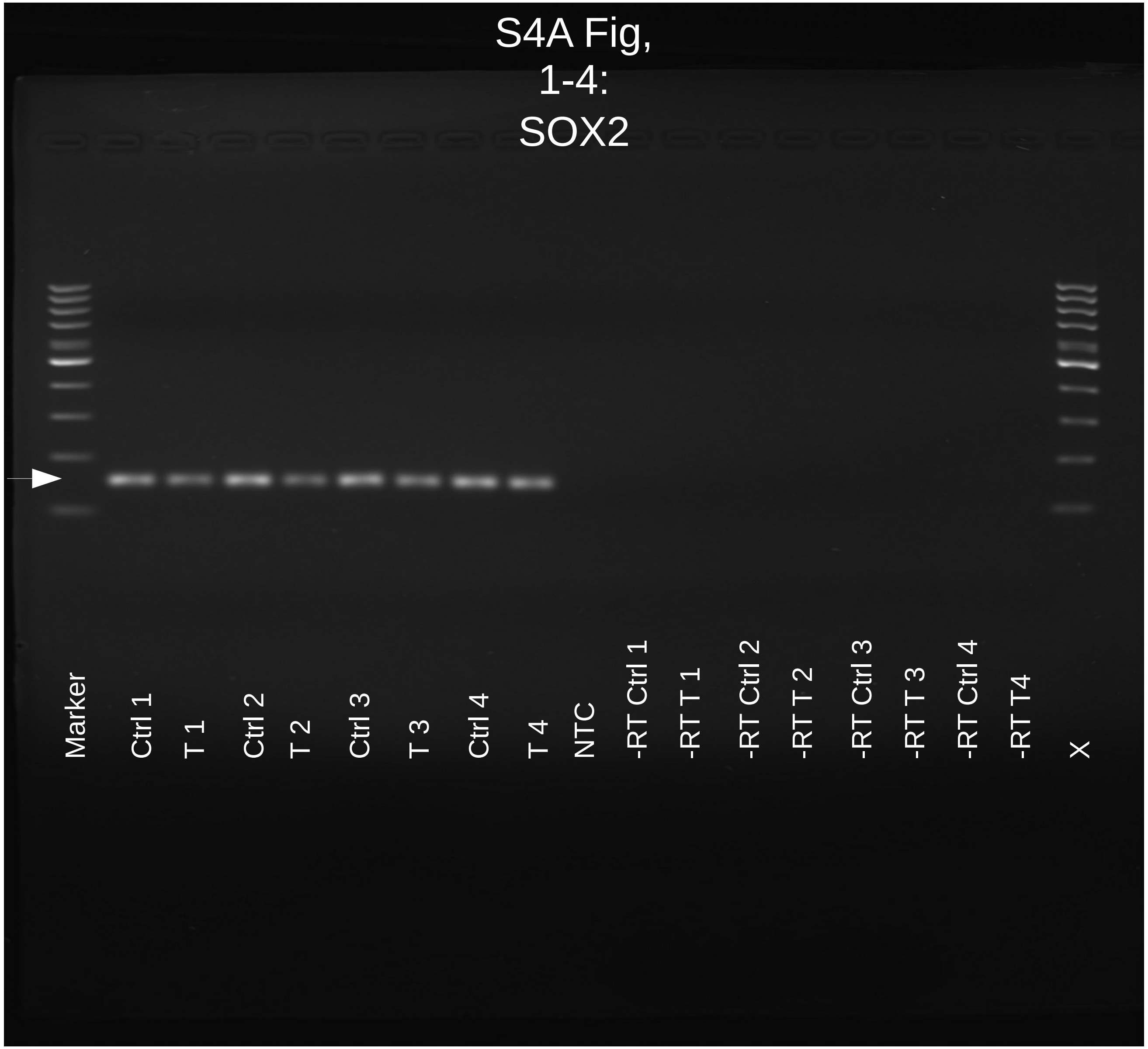
S4A Fig,
1-4:
SOX2
Marker
Ctrl 1
T 1
Ctrl 2
T 2
Ctrl 3
T 3
Ctrl 4
T 4
NTC
-RT Ctrl 1
-RT T 1
-RT Ctrl 2
-RT T 2
-RT Ctrl 3
-RT T 3
-RT Ctrl 4
-RT T4
X

S4A Fig,
1-4:
GAPDH

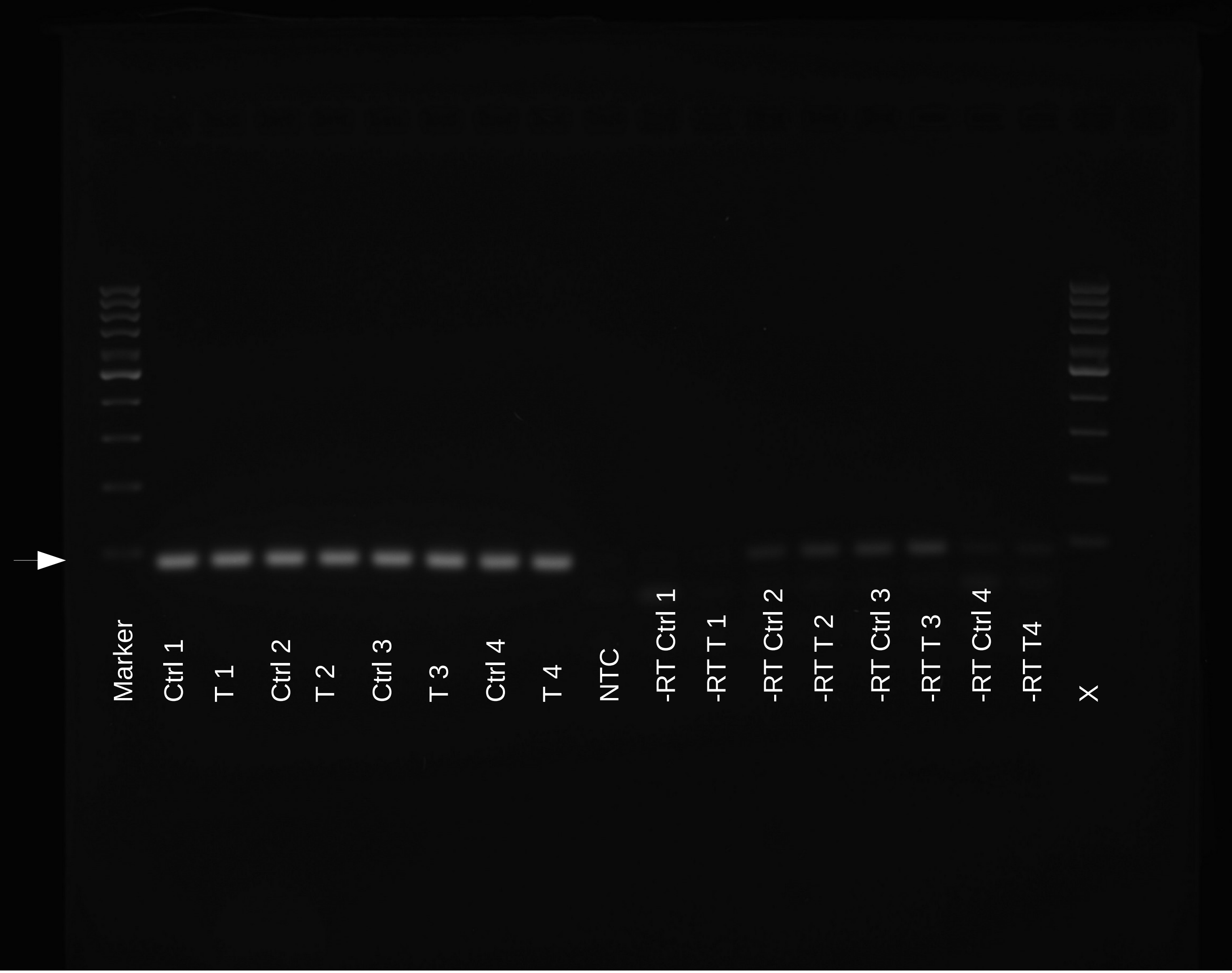

S4A Fig,
5-8:
OCT4A*

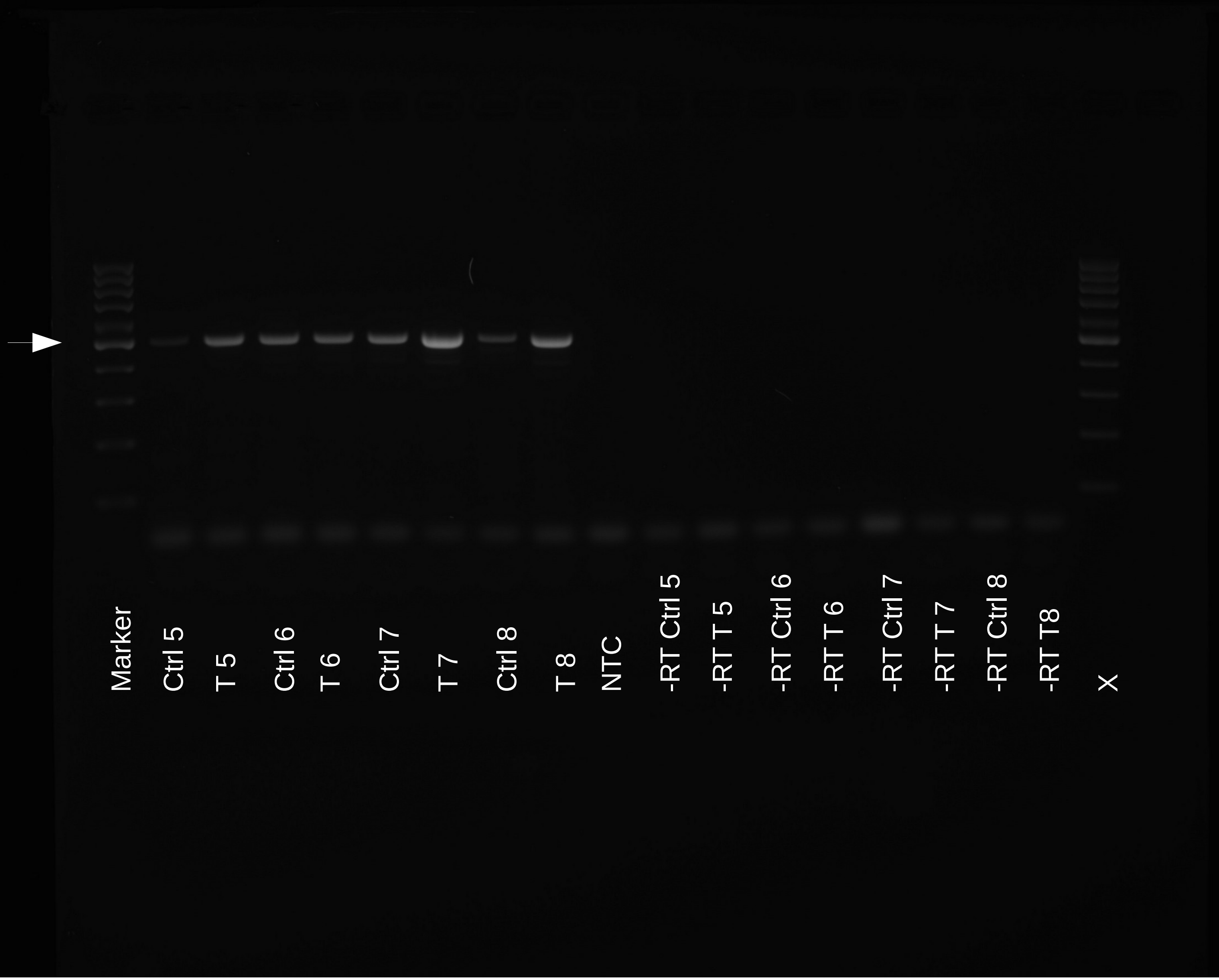

S4A Fig,
5-8:
OCT4B1
OCT4B
OCT4B4

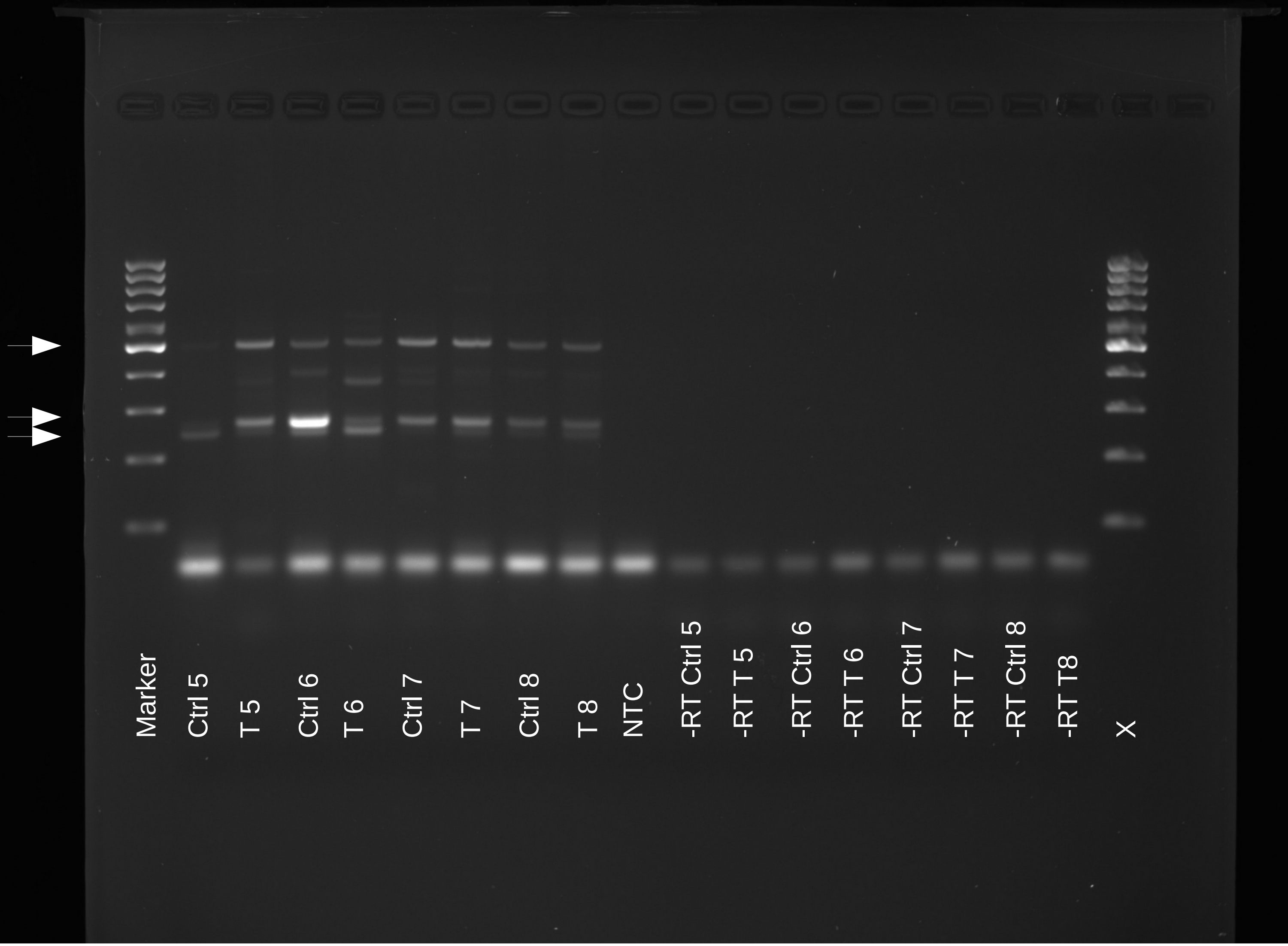

S4A Fig,
5-8:
NANOG1

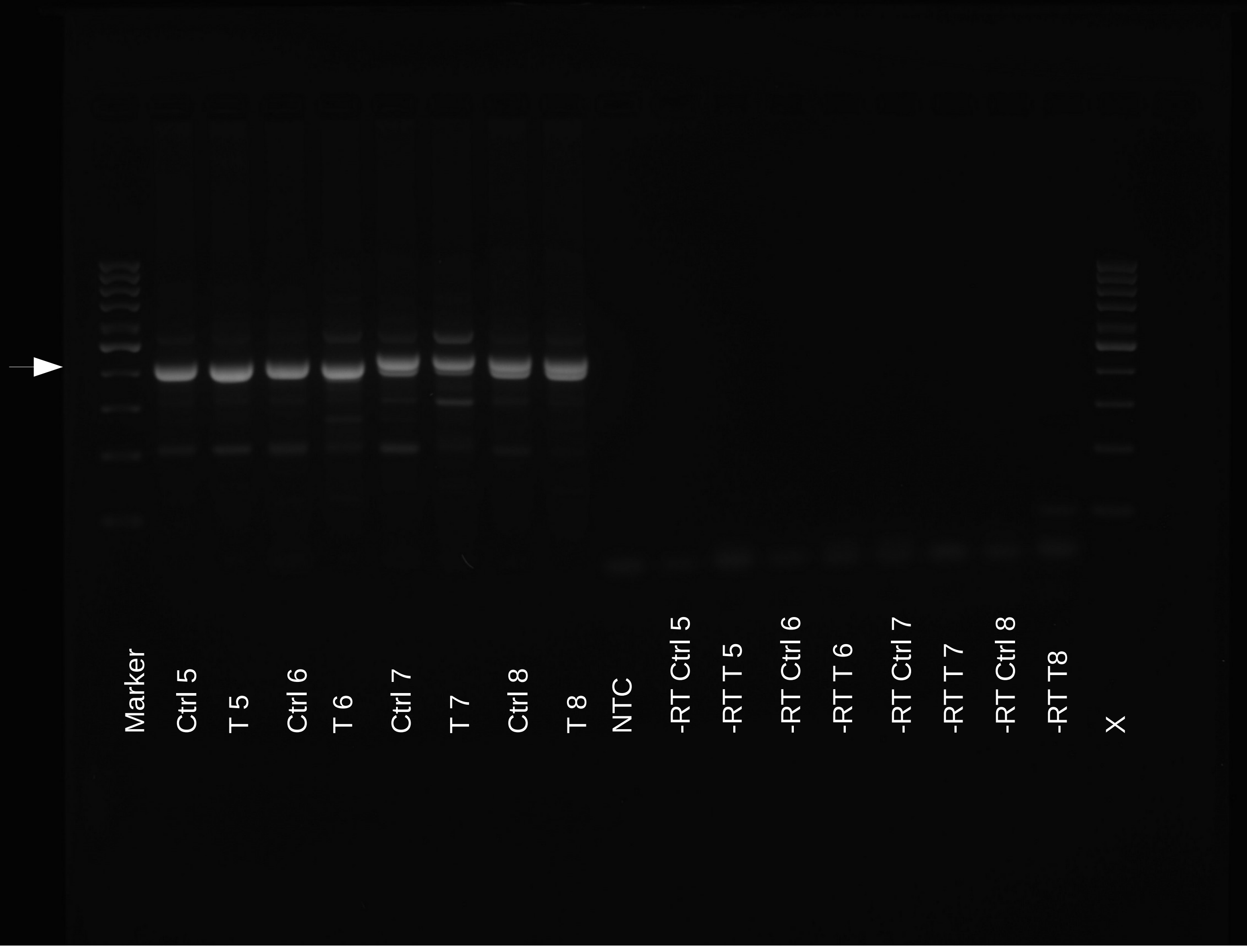

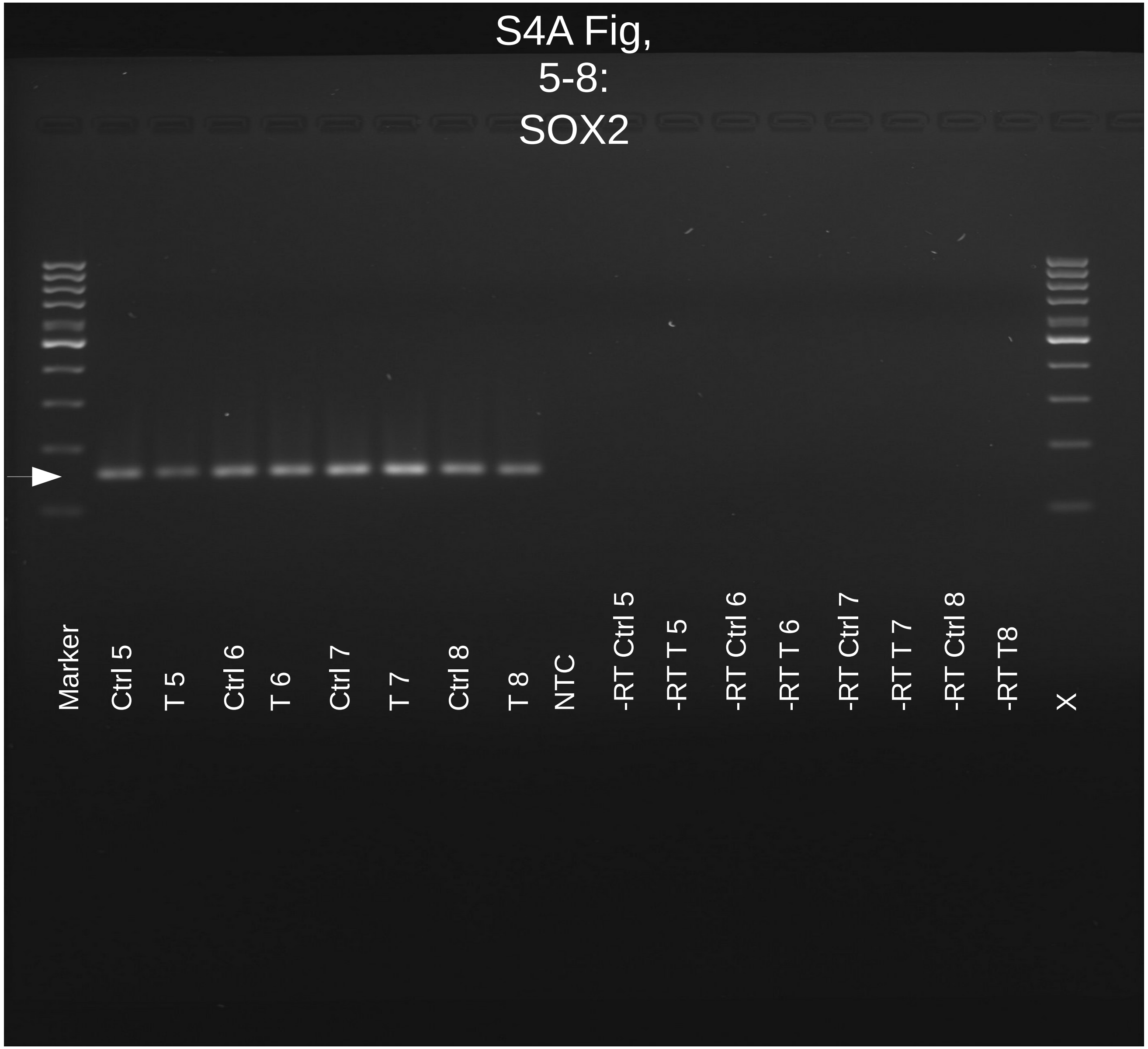
S4A Fig,
5-8:
SOX2
Marker
Ctrl 5
T 5
Ctrl 6
T 6
Ctrl 7
T 7
Ctrl 8
T 8
NTC
-RT Ctrl 5
-RT T 5
-RT Ctrl 6
-RT T 6
-RT Ctrl 7
-RT T 7
-RT Ctrl 8
-RT T8
X

S4A Fig,
5-8:
GAPDH

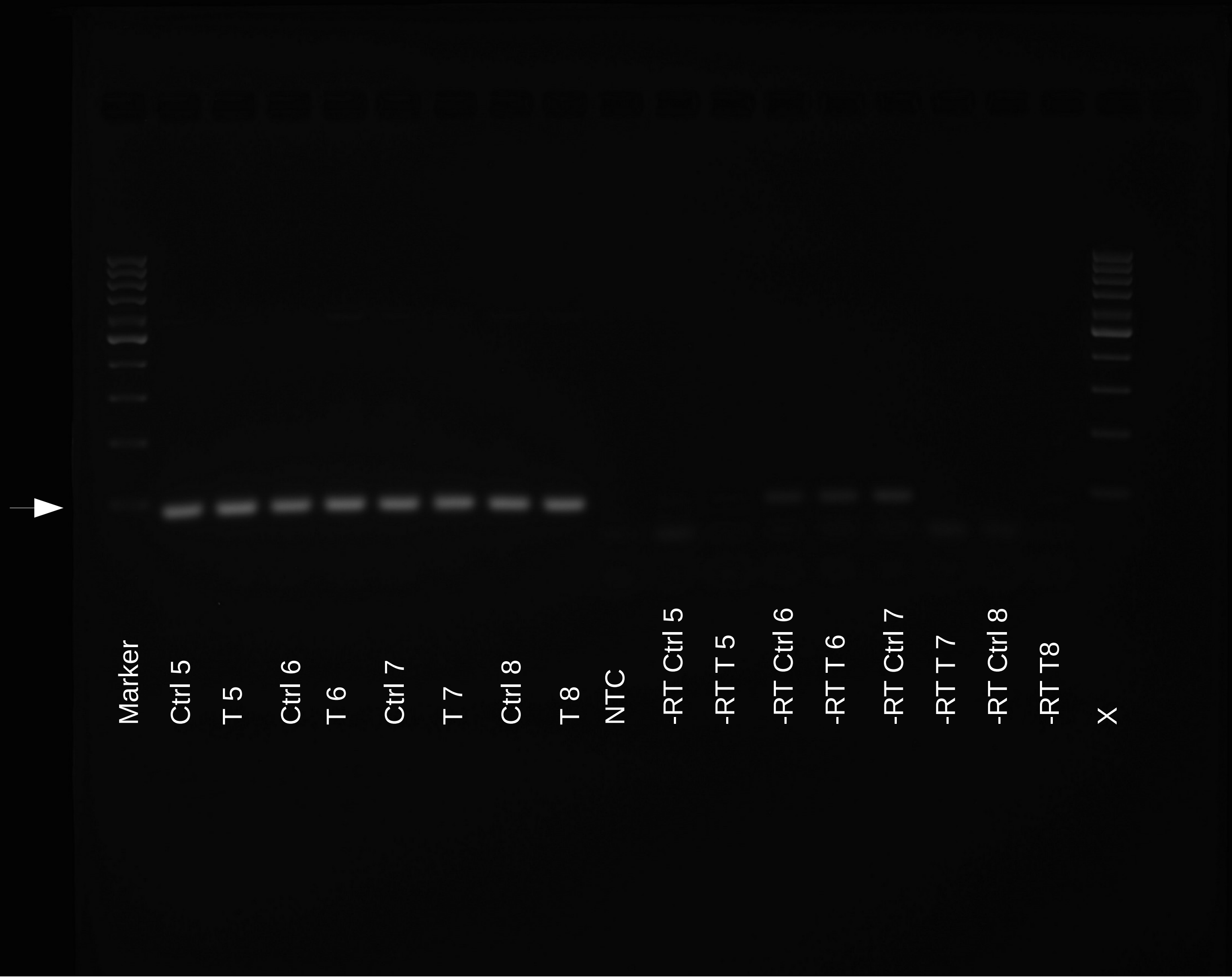

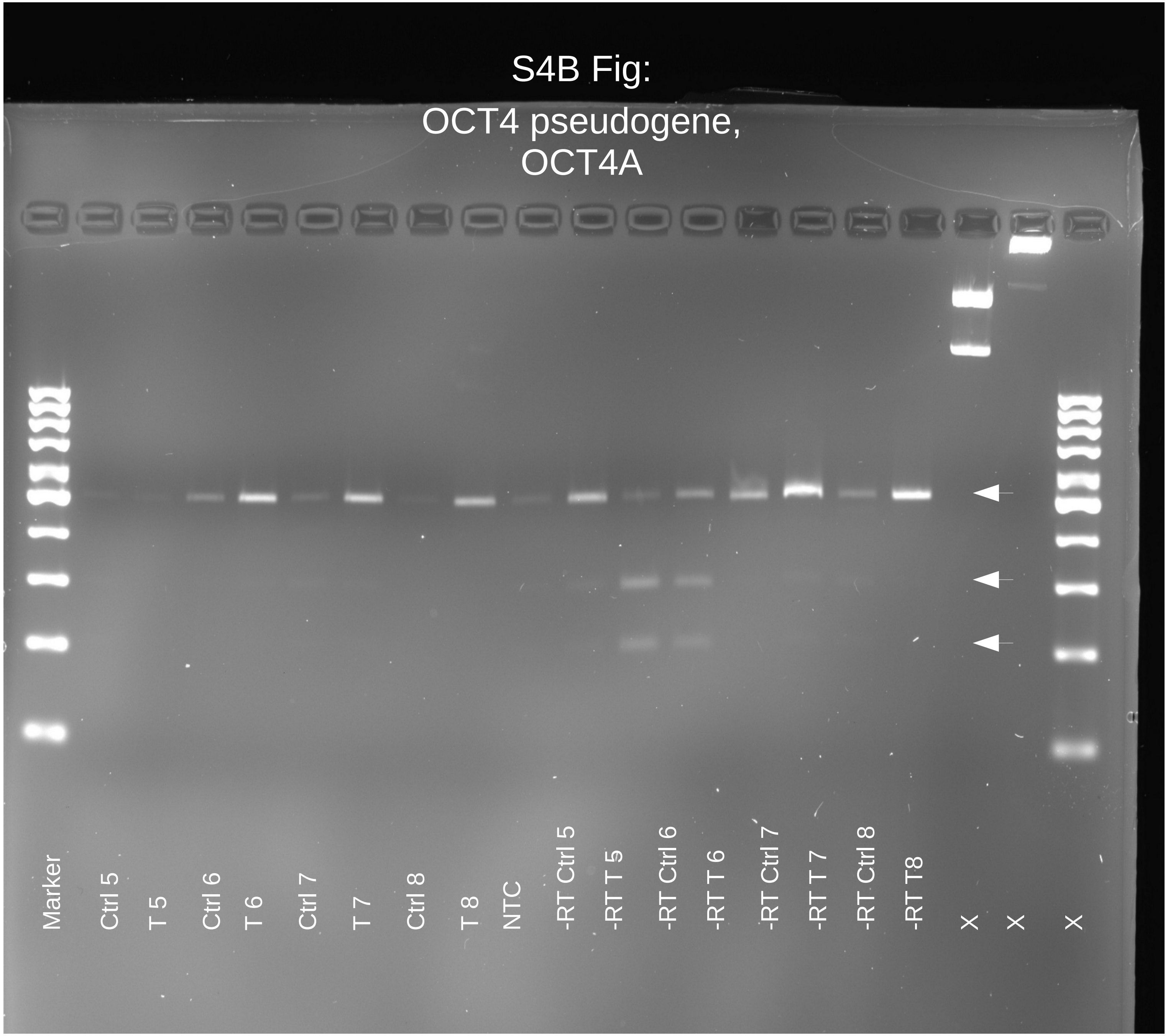

S4B Fig:

OCT4 pseudogene, OCT4A

S5 Fig

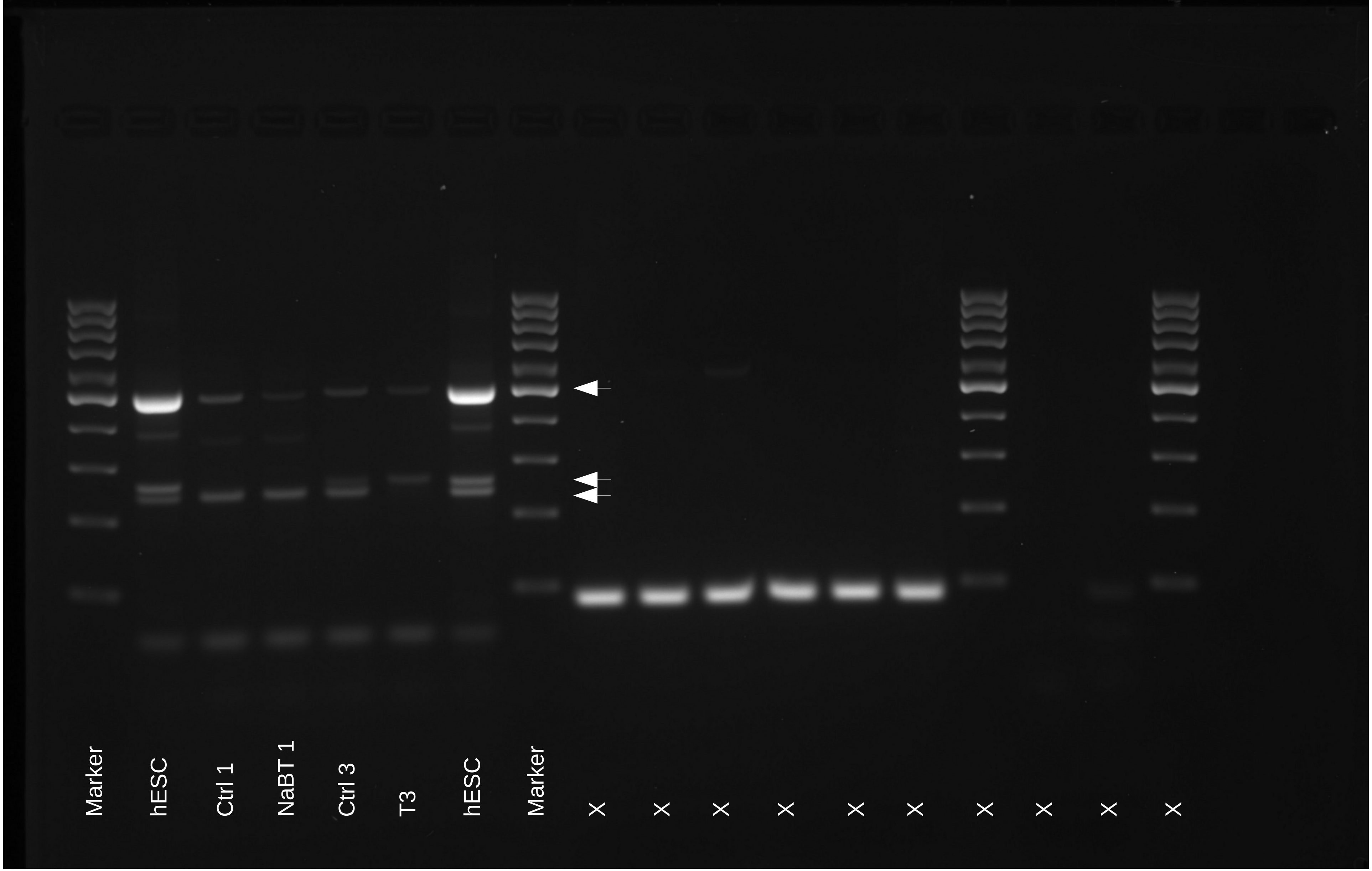

Supplement: S1 Raw images — (PDF) [file pone.0245348.s013.pdf]
